# A Potential Concomitant Sellar Embryonic Remnant-Associated Collision Tumor: Systematic Review

**DOI:** 10.3389/fonc.2021.649958

**Published:** 2021-04-29

**Authors:** Mingdong Wang, Qianhui Fu, Mingjing Song, Zongmao Zhao, Renzhi Wang, John Zhang, Wenbin Ma, Zhanxiang Wang

**Affiliations:** ^1^ Department of Neurosurgery, The First Affiliated Hospital of Xiamen University, School of Medicine, Xiamen University, Xiamen, China; ^2^ School of Pharmacy, Minzu University of China, Beijing, China; ^3^ Institute of Laboratory Animal Science, Chinese Academy of Medical Science and Comparative Medical Center, Peking Union Medical College, Beijing, China; ^4^ Department of Neurosurgery, The Second Hospital of Hebei Medical University, Hebei, China; ^5^ Department of Neurosurgery, Peking Union Medical College Hospital, Beijing, China; ^6^ Physiology Program, Department of Anesthesiology, Neurosurgery, Neurology, and Physiology, Center for Neuroscience Research, Loma Linda, CA, United States

**Keywords:** collision sellar lesions, Rathke cleft cyst, solid sellar lesion, sellar embryonic-remnants lesions, cystic sellar lesions

## Abstract

**Background:**

Diagnosing the well-known concomitant Rathke’s cleft cyst (RCC) and differentiating it from other sellar lesions are difficult because RCC is and other sellar lesions are closely related and represent a continuum from simple RCCs to more complex lesions. The purpose of this study is to better understand the adeno- and neurohypophysis adjacent to the par intermedia remnants and their role in the origin of the coexistence of these two distinct tumor neoplasias; to assess the incidence in different age groups; to categorize the pathohistological subtype, which can be incorporated in predictive/prognostic models; and finally, to evaluate the current evidence on collision tumors of the sellar embryonic remnant tract in terms of their biological behavior and pathology.

**Methods:**

Utilizing the PubMed database, data were collected from 1920 to 2019. Information about demographics, clinical characteristics, and age was summarized and analyzed by using univariable and multivariable models. The same cell type was observed regardless of whether the tumor was only one type or mixed types, and their histologic patterns were assessed.

**Results:**

The incidence rates were similar among patients stratified into three age subgroups: 40–49 years (24.57%), 50–59 years (19.54%), and older than 60 years (22.98%). We found that various types of sellar lesions, namely, squamous metaplasia (SM) + goblet cells (GC) (HR 46.326), foamy macrophages (FM) (HR 39.625), epithelial cells and multinucleated giant cells or cholesterin (EM) (HR 13.195), a cavernous portion of the right internal carotid artery (CP-ICA) (HR 9.427), epithelial cells with ciliated cuboidal (EC-CC) (HR 8.456), were independently associated with RCC pathological status. These divergent AUCs (0.848 for Hypo as RCC, 0.981 for RCC co PA, 0.926 for CD and CP co RCC) and subtypes of PA (HR 4.415, HR 2.286), Hypo (HR 3.310), CD and CP (HR 2.467), EC and DC and PG and SGR (HR 1.068), coexisting with the risk of a comorbid RCC lesion, may reflect the etiologic heterogeneity of coderivation and the different effects of some risk factors on tumor subtypes. Our analyses suggested that the greatest accuracy was observed for the pituitary adenoma subtype, with an AUC of 0.981 (95% confidence interval [CI]: 0.959–1.005), while the poorest accuracy was observed for aneurysms, with an AUC of 0.531 (95% CI: 0.104–0.958). We separately analyzed and confirmed the above results. Sensitivity analysis revealed no evidence of systematic bias due to missing data.

**Conclusion:**

This study showed that the histopathological changes in patients with sellar embryonic remnant-associated collision tumors showed highly consistent epithelial cell replacement (renewal) (ciliated columnar epithelium to ciliated squamous epithelium to squamous epithelium) or accumulation, and the RCC cyst wall was similar in structure to the tracheobronchial airway epithelium, with progenitor cell characteristics. The collision accuracy between RCC and other tumors (PAs, craniopharyngioma, chordoma, etc.) is different; these characteristics constitute the theoretical basis for the postmigration development of the pharyngeal bursa.

## Background

The anterior limb of Rathke’s pouch contributes to the anterior lobe and forms the posterior lobe of the hypophysis, which is derived from two components: neuroectodermal evagination and oral ectoderm invagination (1, 2). Embryonic remnants of the diverticulum of Rathke’s pouch at the junction between the anterior and posterior lobes (in the area representing the vestigial intermediate lobe) normally regresses and is less distinct in humans but almost always contains small cysts, namely, Rathke’s cleft cysts (RCCs), and the cells lining the cleft are thought to be the origin of various cystic lesions. These cysts are lined with one or multiple layers of epithelial cells; some cysts are lined with cuboidal or columnar ciliated cells, while others are lined with flattened cells (3–5). Consequently, this category of tumor displays considerable variation in cytology, encompassing a range of primitive homologous cells in the midline ectoderm. However, the nature of sellar embryonic remnant-associated collision tumors (S-ErACTs) is not well understood.

A diversity of S-ErACT occurs in humans, including RCC, chordomas (CD), craniopharyngiomas (CPs), dermoid cyst (DC), epidermoid cyst (EC), salivary gland remnants (SGR), and atypical teratoid rhabdoid tumor (AT/RT), which can occur individually or concomitantly. One of the hallmarks of S-ErACT is its sellar neuroendocrine clinical and biological heterogeneity. Previous work has suggested that another variant of RCC elements is nested within a typical papillary or adamantinomatous lesion (6). This is consistent with the basic definition of collision tumors (CTs), which are composed of two neighboring independent neoplasms that coexist with little or no interaction between them (7). It is unclear whether these concomitant tumors arise from remnants of Rathke’s pouch, embryonic remnant cells that exhibit metaplasia in the sellar region, or a possible common origin or other association, such as the induction of stratified squamous epithelial progenitor cells. Most RCCs are within the sellar region between the anterior and posterior lobes, but some extend to the optic chiasm. Strikingly, the dysembryogenetic conditions of the sellar and suprasellar regions are probably underrecognized (8). This persisting embryonal infundibular recess (PEIR) can be misdiagnosed as a sellar lesion; however, it is relatively rare.

The origin of RCCs is unclear, and no systematic genetic and pathology research has been conducted in humans. Rathke’s pouch remnants, an ectodermal diverticulum arising from the foregut, may give rise to Rathke’s cysts. To date, it is still unclear whether these cysts are incidental. However, a high rate of cystic lesions has been noted in autopsy cases (13–33%) (4, 9). Cysts are hypothesized to arise from oral ectoderm cells of the pharyngeal duct that fail to degenerate. This idea is supported by the ability of the oral epithelium or pharyngeal bursa to grow (4, 10). RCC was observed in 1.2% of patients between the ages of 1 and 4 years (11). These patients are different when compared with children of the same age or adult. RCC patients display a slow-growing in adulthood (12). The incidence of concomitant occurrence is unclear, but estimates range from 0.51 to 3.55% of intrasellar lesions (13). Some RCC patients have prolactinoma, Cushing’s disease, Pluri-hormonal and double adenoma (PIA), non-functioning pituitary adenoma (NFPA), or transitional cell tumor (TCT) of the pituitary or pituitary oncocytoma (14).

We propose that pharyngeal bursa migration and stratified squamous epithelial progenitor cells can be caused by the continued growth of the nasopharynx, a pharyngeal bursa appearing at the anchoring point of the notochord and overlying endoderm, and stromal cell-derived induction activity in residual embryonic tissue that could induce subsequent development of the remnant diverticulum of Rathke’s pouch. Although RCC and pituitary neuroendocrine tumors (PitNETs) or other benign sellar lesions rarely occur together, the origin and histopathology remain controversial in the literature. However, many factors affect the pathogenic process of concomitant disease due to the risk of recurrence. These embryological changes prompted us to examine the histopathological and clinical presentation of pituitary function to determine whether coderivation (the identification of continually changing pathological tissues with potential stem cell characteristics) was concomitantly associated (continuum) with both diseases in the sellar region.

This study aimed to clarify the correlation between the stratified squamous epithelium cells of RCC and the characteristics of pluripotent stem cells (stratified squamous epithelial progenitor cells) and coexisting sellar lesions with a focus on the relationship between the main factors affecting the development and growth of residual embryo tissue and pathological changes.

## Methods

We performed a literature search of the MEDLINE databases, Wiley online library, Science Direct, and Web of Science from March 1, 1920, through April 1, 2019. Searches of PubMed over the past 99 years were conducted using terms related to RCC. We included case series (case reports) on patient characteristics, interventions and comparisons, and randomized controlled trials (RCTs) in English or Chinese. All authors reviewed the abstracts to assess eligibility. The included studies fulfilled the following requirement: All studies reporting RCC were included. The systematic review followed preferred reporting items for systematic reviews and meta-analyses (PRISMA) guidelines (see **Supplementary Appendix-1** pp 1).

### Patient and Study Eligibility

We included patients and studies that met the following inclusion criteria (**Supplementary Appendix-1**: The diagnosis standard of pituitary adenoma): 1) We included observational and treatment (surgery plus radiotherapy) studies on coexisting RCC and sellar region lesions that contain data about the population-based prevalence of symptomatic RCC coexisting with the sellar lesion (the cooccurrence of the two diseases is the basic feature). 2) We included patients with a diagnosis of RCC coexisting with sellar lesion based on suggestions from qualified neurosurgeons and radiologists, confirmatory imaging evidence from CT, MRI, DSA, CTA, and MRA, observations during operation, and histological examinations. 3) Cross-checking of references was performed until no new articles were identified. Available full-text articles written in English or Chinese. The study had to be retrospective by definition. 4) For an explanation of cases without modern imaging in the 1920s and 1970s and the primary basis of pathological diagnosis, see the appendix (pp 4). For the classification of tumors of the pituitary gland, see the **Supplementary Information** (pp 7,8).

We excluded studies based on the following: 1) abstracts from conferences, full texts without raw data, duplicate publications, letters, or reviews (no case reports); or 2) RCC coexisting with sellar lesions that was diagnosed based on objective imaging, observations in operation, histological examinations, and medical records but only by imaging examination (lack of laboratory or endocrinology examination or self-reported cases).

### Data Analysis (Data Extraction, Quality Assessment)

Two investigators independently extracted relevant data using a standardized from: publication year, origin of study (first author), type of study (study design, prospective, retrospective, single-center, multi-center), size of study population, mean age, rang of patients, hormone level, clinical imaging, pituitary function, Clinical diagnosis, surgical treatment, histopathological subtypes, following-up time, survival and died. Two authors cross-checked the results. Based on these data included 67 articles, 7-by-8 tables were constructed from each study based on raw data. Each study was rated with regard to the following domains: patient selection, pituitary function, hormone level, therapeutic method, following-up, reference standard, timing, the risk of bias, and the results were discussed by all authors.

### Risk of Bias Assessment

The Cochrane risk of bias tool was used to assess the construct validity for preclinical studies, the clinical generalizability of the experimental conditions, and the seven domains involved.

### Statistical Analysis

We first used R version 3.4 (statistic package studio) to implement the random forest algorithm and obtain the important categorical variable score (see the **Appendix—**67meta-analyses were identified, pp 10). The association between unitary factors and clinical surgery was regarded as the outcome. Next, we performed several *post hoc* sensitivity analyses. A Cox proportional hazards model and Bayesian analysis for multivariable analysis were applied to the variables. The Kaplan-Meier (KM) method for overall survival (OS) and relapse-free survival (RFS) analysis was performed for six subgroups of patients.

The receiver operating characteristic (ROC) curve was used to evaluate the performance of the constructed models. ROC comparison analysis was performed to assess significant differences in AUCs by using the method developed by DeLong et al. (15).

## Result

### Patient Demographics

Sixty-seven studies met our pre-established inclusion criteria (for our case, see supplementary, pp 10) (**Supplementary Data-Figure S1**). Patient demographic, clinical and histopathological data are shown in **Tables 1**, **2**, **3**, and **4**. Among the 118 studied patients (including our own patients), 43 (36.44%) were male, and 75 (63.55%) were female. The age characteristics of patients with RCC concomitant with various sellar lesions are summarized in **Table 1**. The rates of various sellar lesions were (male, female) (36.78%, 63.21%), (31.25%, 68.75%), (50%, 50%), and (20%, 80%).

**Table 1:** The age characteristic of RCC concomitance sellar lesion.

Parameter (No. Cases)	RCC co PA Subtypes—other and Acromegaly N = 87	RCC as Hypophysitis N = 16	RCC co Aneurysm N = 4	RCC co Chordoma and Cran and other lesion N = 11	Total: N = 118
Gender (%)					
Male	32 (36.78%)	5 (31.25%)	2 (50%)	4 (20%)	43 (36.44%)
Female	55 (63.21%)	11 (68.75%)	2 (50%)	7 (80%)	75 (63.55%)
Age (%)					
0–11y	1 (1.14%)	1 (6.25%)	0	1 (9%)	3 (2.54%)
12–19y	2 (2.29%)	2 (12.5%)	0	3 (27.27%)	7 (5.93%)
20–29y	16 (18.39%)	3 (18.75%)	0	–	19 (16.10%)
30–39y	21 (24.13%)	2 (12.5%)	0	–	23 (19.49%)
40–49y	20 (22.98%)	4 (25%)	1(25%)	4 (36.36%)	29 (24.57%)
50–59y	14 (16.09%)	2 (12.5%)	0	1 (9%)	17 (19.54%)
≥60y	13 (14.94%)	2 (12.5%)	3(75%)	2 (18.18%)	20 (22.98%)
					
F*	7.606	12.714	5.581	2.245	
P* Value	0	–	0	0.044	

RCC, Rathke’s cleft cysts; PA, pituitary adenoma; co, co-existent; as, associated with. Other: include Lactotroph adenoma, Plurihormonal adenoma, non-functioning pituitary adenoma, adrenocorticotropin adenoma, Thyrotroph adenoma. Other lesion: include Epidermoid Cyst, Dermoid Cyst, pituitary granulomatosis, Salivary, Salivary gland remnants. F* P*: P value from post-hoc test for categorical variables. Hypop, Hypophysitis; Chor, Chordoma; Acro, Acromegaly; Cran, Craniopharyngioma.

**Table 2:** RCC coexistent pituitary adenoma subtype and histopathological features and immunohistochemistry.

Adenoma type (No. Cases)	Morphological (%)	Immunohistochemistry (IHC) (%)	HR (95% CI) P*	P*
Total PA co RCC n=87				
LA+ CA-CCC	29 (33.33%)	31 (47.69%)	2.118 (1.311–3.422)	0.002
TA+ CA-CCC	1 (1.14%)	4 (6.15%)	1.763 (0.243–12.805)	0.575
PIA+ CA-CCC/SSEC	6 (6.89%)	7 (10.76%)	1.524 (0.651–3.564)	0.332
NFPA+ CA-CCC/CIC	14 (16.09%)	18 (27.69%)	2.049 (1.101–3.814)	0.024
ACTHA+ CA-CCC	8 (9.19%)	8 (12.3%)	2.652 (1.235–5.696)	0.012
Other + CA-CCC	9 (10.34%)	11 (16.92%)	2.719 (1.314–5.626)	0.007
SA(DG)+ CA-CCC	2 (10%)	–	0.902 (0.125–6.485)	0.918
SA+ CA-CCC	17 (85%)	–	2.448 (1.441–4.158)	0.001
Gangl	1 (5%)	–	0.506 (0.070–3.651)	0.499

Neg, negative; Co, coexistent; As, associated with; DG, densely granulated. SG, Sparsely granulated. SA, Somatotroph adenoma; TA, Thyrotroph adenoma; LA, Lactotroph adenoma; PIA, Plurihormonal adenoma; NFPA, non-functioning pituitary adenoma; ACTHA, adrenocorticotropin adenoma; CA-CCC, ciliated columnar cell or monolayer of cuboidal; SSEC, stratified squamous epithelium cell; CIC, chronic inflammatory cells; IC, inflammatory cells (mainly lymphocytes and plasma); HR, hazard ratio; CI, indicates confidence interval; Gangl, gangliocytomas; Other, gonadotroph adenoma with craniopharyngioma, Null-cell adenoma. P*: P value. Other: include gonadotroph.

**Table 3:** RCC coexistent various type other sellar lesion and histopathological features and immunohistochemistry.

Coexistence sellar lesion category (No. Cases)	Morphological (%)	Immunohistochemistry (IHC) (%)	HR (95% CI) P*
RCC as Hypophysitis n = 16			
SM+GC/FM +CA-CCC+IC	4(25%)	–	46.326 (8.080–265.600) 0.000
No-FM+CA-CCC+IC	3(18.75%)	–	39.625 (6.369–246.521) 0.000
EM+CA-CCC+IC	9(56.25%	–	13.195 (2.152–80.917) 0.005
–	-	–	
RCC co Aneurysm n = 4			
CP-ICA+ CA-CCC/SSEC	1 (25%)	1 (25%)	9.427 (1.225–72.555) 0.031
ACA+ CA-CCC/SSEC	1 (25%)	1 (25%)	9.427 (1.225–72.555) 0.031
A-A1-C A+ CA-CCC/SSEC	1 (25%)	1 (25%)	9.427 (1.225–72.555) 0.031
A-com A+ CA-CCC/SSEC	1 (25%)	1 (25%)	3.111 (0.425–22.772) 0.264
RCC co Chordoma and Craniopharyngioma n = 5			
EITC+CA-CCC/SSEC	3 (60%)	3 (60%)	0.728 (0.230–2.304) 0.589
mMC+CA-CCC	2 (40%)	2 (40%)	3.244 (0.782–13.466) 0.105
EMA	–	–	–
RCC co Other sellar lesion n = 6			
EC-CC+ CA-CCC/SSEC	1 (16.66%)	1 (16.66%)	8.456 (1.108–64.522) 0.039
RTE+ CA-CCC/SSEC	1 (16.66%)	1 (16.66%)	–
Xantho+CA-CCC/SSEC	1 (16.66%)	1 (16.66%)	3.275 (0.447–24.008) 0.243
CIC + CA-CCC/SSEC	1 (16.66%)	1 (16.66%)	5.194 (0.698–38.679) 0.108
SGR+ CA-CCC/SSEC	1 (16.66%)	1 (16.66%)	2.560 (0.351–18.666) 0.354
HA+ CA-CCC/SSEC	1 (16.66%)	1 (16.66%)	0.633 (0.88–4.560) 0.650

CA-CCC, ciliated columnar cell or monolayer of cuboidal; SSEC, stratified squamous epithelium cell; EITC, epithelioid-like tumor cells; SM, squamous metaplasia; GC, goblet cells; MC, mucous cells and basal cells; EC-CC, epidermoid cyst with ciliated cuboidal; FM, foamy macrophages; EM, epithelial cells and multinucleated giant cells or cholesterin; RTE, respiratory type epithelium; Xantho, xanthogranulomatous, CIC, chronic inflammatory cells; SGR, salivary gland remnants; HA, hematoma; CP-ICA, cavernous portion of the right internal carotid artery; ACA, anterior cerebral artery; AcomA, anterior communicating artery aneurysm; IC, inflammatory cells (mainly lymphocytes and plasma); HR, hazard ratio; CI, confidence interval. Other sellar lesion: include Epidermoid Cyst, Dermoid Cyst, pituitary granulomatosis, Salivary, Salivary gland remnants. P*: P value.

**Table 4:** Total surgical data and follow-up sellar lesion and pituitary adenoma subtype.

Factors parameters	RCC co PA Subtypes—other and Acromegaly N = 87		RCC as Hypophysitis N = 16	RCC co Aneurysm N = 4	RCC co Other sellar lesion-N = 11	
	RCC co Subtype PA—other N = 67	RCC co Subtype PA—Acromegaly N = 20			Chordoma and Craniopharyngioma N = 5	Other sellar lesions N = 6
HR	4.415	2.286	3.31	0.937	2.467	1.068
(95% CI)	(1.803–10.808)	(0.886–5.898)	(1.230–8.907)	(0.263–3.340)	(0.736–8.269)	(0.299–3.807)
P*	0.001	0.087	0.018	0.92	0.143	0.92
AUC	0.981		0.848	0.531	0.926	
(95% CI)	(0.959–1.005)		(0.687–1.008)	(0.104–0.958)	(0.806–1.046)	
Preoperative-PF (%)						
PRL↑	32 (47.76%)	–	–	–	1 (20%)	–
GH↑	4 (5.97%)	10 (50%)	–	–	–	–
ACTH↑	5 (7.46%)	–	1 (6.25%)	–	1 (20%)	–
DI/+Hypo	–	1 (5%)	3 (18.75%)	–	–	–
FSH/LH/T TSH/FSH	2 (2.98%)	–	–	–	1 (20%)	–
Unknown	2 (2.98%)	–	1 (6.25%)	–	–	1 (20%)
Hypo/PHP	6 (8.95%)	9 (45%)	–	–	3 (60%)	4 (60%)
Normal	2 (2.98%)		10 (62.5%)		1 (20%)	1 (20%)
	14 (20.89%)		2 (12.5%)		1 (20%)	
Postoperative-PF (%)						
DI/+Hypo/+AI	6 (8.95%)	2 (10%)	5 (31.25%)	–	1 (20%)	1 (20%)
Hypo/+TD	-	–	–	1 (25%)	–	1 (20%)
PHP	3 (4.47%)	–	4 (25%)	–	1 (20%)	2 (30%)
Normal	56 (83.58%)	13 (65%)	6 (37.5%)	3 (75%)	3 (60%)	2 (30%)
Unknown	2 (2.98%)	5 (25%)	1 (6.25%)			
Therapeutics (%)						
TSS	60 (89.55%)	19 (95%)	15 (93.75%)	–	4 (66.67%)	4*(70%)
Cr/ ± CE	5 (7.46%)	–	1 (6.25%)	4 (100%)	2 (33.33%)	2 (30%)
Conservative	1 (1.49%)	1 (5%)	–	–	–	–
REPL-T	2 (2.98%)	–	–	–	–	–
Follow-up time (%)						
3–12 months	9(14.43%)	5 (25%)	–	3 (75%)	–	1 (20%)
1–3 years	13 (19.40%)	2 (10%)	5 (31.25%)	1 (25%)	1 (20%)	2 (30%)
3–7 years	7 (10.44%)	1 (5%)	1 (6.25%)	–	–	–
Uncertain	34 (50.75%)	12 (60%)	10 (62.5%)		4 (80%)	3 (50%)
Recurrence	2 (2.98%)					
Died	2 (2.98%)					

PF, Pituitary Function; co, coexistent; as, associated with; other, pituitary adenomas of other types except growth hormone adenomas type; Hypo, Hypopituitarism; AI, adrenal insufficiency; TSS, trans-sphenoidal surgical; CCC, Ciliated columnar cells; SSC, stratified squamous cells; DI, diabetes insipidus; PHP, panhypopituitarism; TD, thyroid dysfunction; Cr, Craniotomy; CE, coil embolization. Other sellar lesion：Epidermoid+ Dermoid+Salivary gland remnants+granulomatosis+pituitary abscess+pituitary apoplexy. 4*, one of the1case had 5time TSS; HR, hazard ratio; CI, indicates confidence interval; REPL-T, Replacement therapy. P*, P value. AUC, area under the curve. Uncertain, include lost follow-up; follow-up time.

### Clinical Presentation and Risk Parameters

The clinical and biologic characteristics at diagnosis of the 118 patients in the analytic cohort with an assigned primary lesion site are listed in **Table 4**. Among them, 45.76% had hormone hypersecretion, 5.93% had hypo-TSH/FSH/LH, 15.25% had diabetes insipidus hypopituitarism/panhypopituitarism, 22.88% had diabetes insipidus (DI) or hypopituitarism postoperatively, and 1.69% had new-onset disease after tumor resection.

A total of 102 (86.44%) of the 118 patients in the six subgroups underwent transsphenoidal surgery (TSS): 11.86% underwent craniotomy, 1.69% received replacement therapy, and 1.69% received conservative therapy. A total of 2.98% of the patients experienced recurrence, 46.61% completed a clearly outlined follow-up period, 53.39% were considered to have an unclear/uncertain follow-up time or follow-up was not described, 1.69% patients died, and 98.31% patients survived.

Patients in with coexisting disease in different age subgroups and with different pathological characteristics have a higher degree of risk than other patients.

### Stratified Identification Analysis of the Incidence of RCC Associated With Various Types of Sellar Lesions

The incidence of RCC associated with various types of sellar lesions across different age subgroups are summarized in **Table 1**. The rates of coexistence with RCC were 5.93% in the 12- to 19-year-old age groups and a mere 2.54% in the 0- to 11-year-old age groups. The 40–49-year-old, 50–59-year-old, and ≥60-year-old subgroups had rates of coexistence of 24.57, 19.54, and 22.98%, respectively.

**Figure 1:**
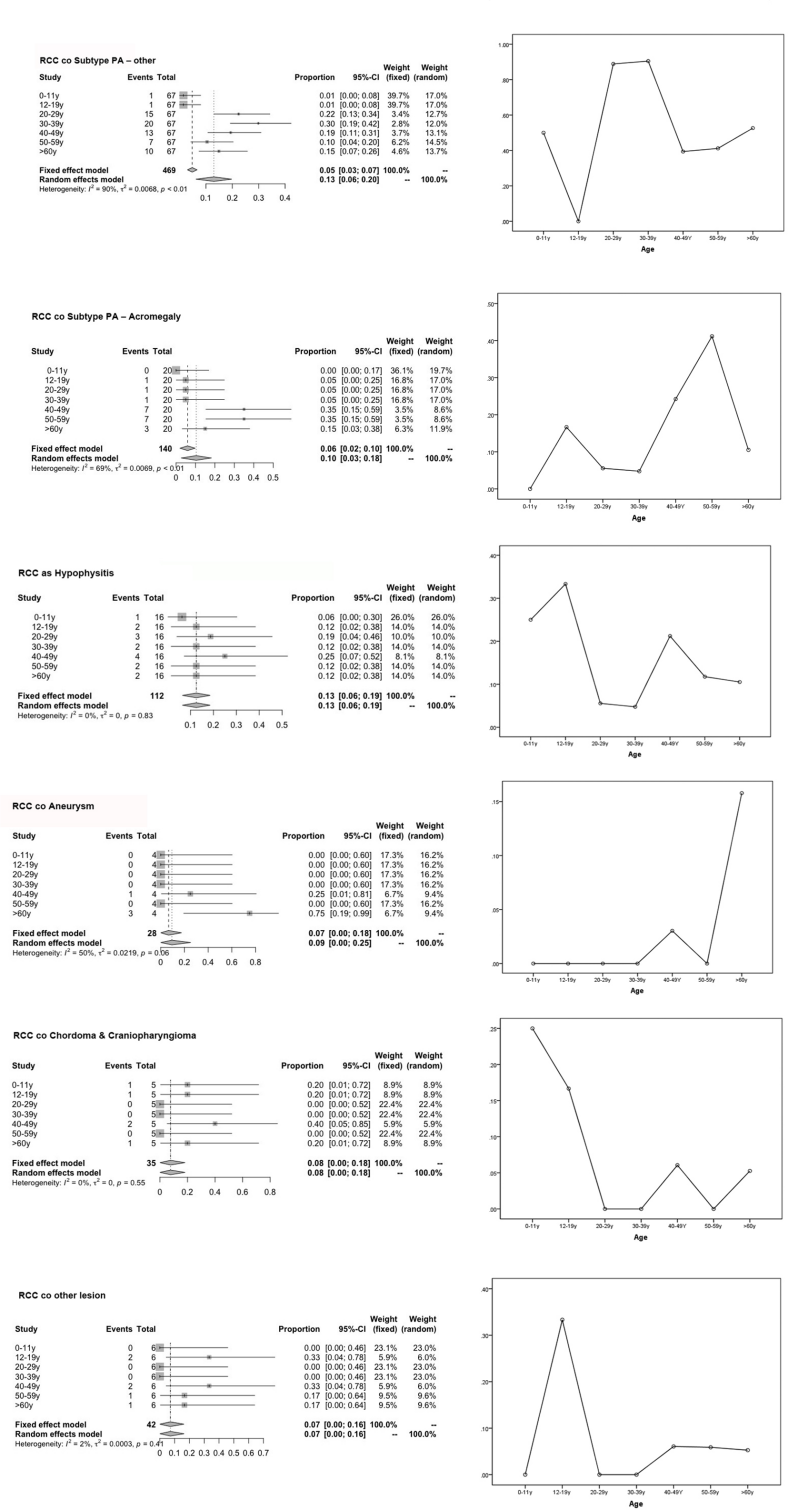
Forest plot for the subgroup analysis of various population-based age groups and risk of age graph. This graph compares the risk of various subgroups (represented by combined RCC with sellar lesion) in each age group and the degree of merger risk within the age group (30–39 y, 50–59 y) calculated using our predictive model.

Notably, the univariate subgroup analysis for age showed that most patients in the 20- to 59-year-old group had the highest incidence rates among all age groups, with tumors that develop over time and remain subclinical until later in life (3). The validation of the three prediction models was performed by assessing the differences in the age groups in terms of the cooccurrence of various types of diseases, along with categorical data (**Table 1**). **Figure 1** displays the most significant hazard of various types of sellar lesions associated with high *vs.* low exposure by age. The association between age and the cooccurrence of various types of sellar diseases varied across age groups and residual embryonic tissues. Regarding age variation, the coexisting risk among patients in the 30–39-year-old, 50–59-year-old, 12–19-year-old, and ≥60-year-old subtypes was greatest for the PA subtype, somatotroph (acromegaly acro) subgroup, Hypo subgroup, and aneurysm subgroup.

Among the other sellar subtypes, the histologic evaluation revealed two types of lesions: respiratory-type epithelium and stratified squamous epithelium cells and ciliated columnar cells (**Table 3**).

### Clinicopathologic Features of Lesion Tissue From Patients With S-ErACT

**Tables 2** and **3** show the significance of each clinicopathologic factor used to identify RCC and various sellar lesions in the 118 patients with the corresponding HR. It is worth noting that the incidence rates (IRs) of squamous metaplasia (SM)/or stratified squamous epithelium cells (SSECs) show different ranges (1.46–22.46%) for different subtypes (RCCs with various sellar lesions and aneurysms) and an increased risk of recurrence.

These lesions (RCC co PA) have been found to contain ciliated columnar cells or monolayers of cuboidal cells and show different incidence rates (IRs), ranging from 0.02 to 27.62%, suggesting that the resected lesion met the criteria of RCC, with extensive squamous metaplasia possessing apical ciliated columnar epithelium and interspersed goblet cells (16). There were inconsistencies in the surgical modalities due to the long treatment era studied.

The analysis of 31 patients with various coexisting types of sellar lesions is presented in **Table 3**. Here, SM+ goblet cells (GC) (HR 46.326, 95% CI 8.080–265.600 P = 0.000), foamy macrophages (FM) (HR 39.625, 95% CI 6.369–246.521 P = 0.000), epithelial cells, and multinucleated giant cells (EM) (HR 13.195, 95% CI 2.152–80.917 P = 0.005), epithelial cells with ciliated cuboidal (EC-CC) (HR 8.456, 95% CI 1.108–64.522 P = 0.039), and CP-ICA (HR 9.427, 95% CI 1.225–72.555 P = 0.031) had a highly significant association. A high-risk pathological type can increase the risk of coexistent RCC and was found to be independently associated with RCC pathological status to various degrees.

### Validation of the Collision Accuracy of Different Subtypes by ROC Curve Analysis in an Independent Cohort of S-ErACT

**Figure 2:**
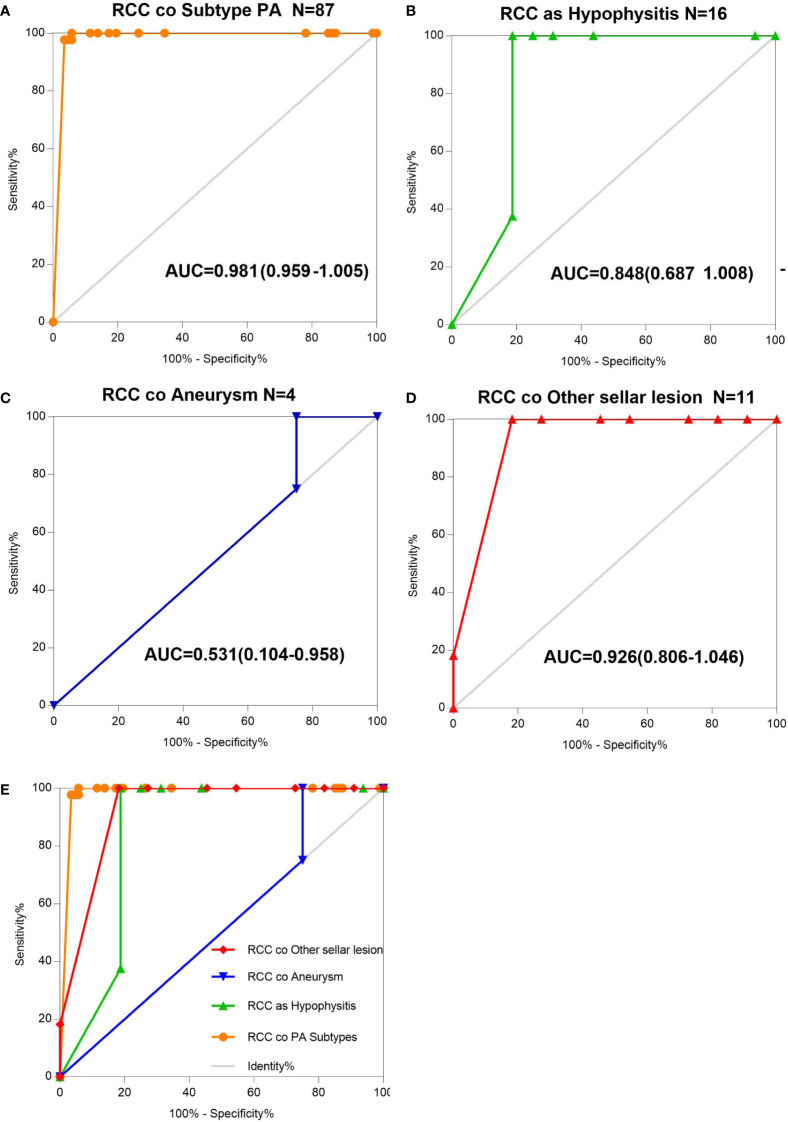
AUC determination using receiver operating characteristic (ROC) curves. **(A)** Information on the results of age group value markers and RCC coexisting with pituitary adenoma subtype, assessed by the AUC. **(B)** Information on the age group value markers and RCC coexisting with hypophysitis, assessed by the AUC. **(C)** Information on the age group value markers and RCC coexisting with aneurysm, assessed by the AUC. **(D)** Information on the age group value markers and RCC coexisting with other sellar lesions, assessed by the AUC. **(E)** RCC and various sellar lesions in 118 patients classified into five groups according to different sellar lesions. Total ROC plot.

The AUCs derived from this analysis equaled 0.848 (95% CI 0.687–1.008) for the hypophysis associated with RCC, 0.981 (95% CI 0.959–1.005) for subtype PA, and 0.926 (95% CI 0.806–1.046) for other sellar lesions (**Figure 2**), and there were marginally significant differences among the six sellar lesion subtypes. The results of the S-ErCT subtype prediction by the ROC curve analysis model with existing unique clinicopathologic features are shown in **Table 4**. The accuracy rate of subtype diagnosis was 89.83%, with the highest accuracy rate achieved for the PA subtype (93.94%). The accuracy rates for hypophysitis, aneurysm, and other sellar lesions were 82, 75.75, and 81.82%, respectively.

To assess the utility of these clinicopathologic feature parameters as diagnostic tools, we performed ROC curve analysis. The ROC curve shows [ciliated columnar cell or monolayer of cuboidal (CA-ccc)] a sensitivity of 89.93% and a specificity of 100%.

### Factors Associated With S-ErACT Formation Included RCC and Various Sellar Lesions and Clinical Characteristics

As presented in **Table 4**, all four subtypes of S-ErACT were significantly associated with RCCs and with all types of PAs (HR 4.415, 95% CI 1.803–10.808, P = 0.001, HR 2.286, 95% CI 0.886–5.898, P = 0.087), Hypo (HR 3.310, 95% CI 1.230–8.907, P = 0.018), CD and CP (HR 2.467, 95% CI 0.736–8.269, P = 0.143), EC and DC and PG and SGR (HR 1.068, 95% CI 0.299–3.807, P = 0.920) coexisting with RCC. An additional analysis was performed to assess the correlations between the clinical features and embryonic remnant-associated tumors of the sellar region in patients with collision lesions. As indicated in **Table 4**, concerning the association of RCC coexisting with all types of sellar lesions by specific subtypes, no difference was observed, regardless of whether CA-ccc and SSEC or GA and SA were present. Among embryonic remnant-related lesions, intermediate lobe-located lesions were associated with the pituitary and CP compared with the suprasellar septum or posterior lobe. RCC and sellar lesions showed an association with coexisting lesions.

**Figure 3:**
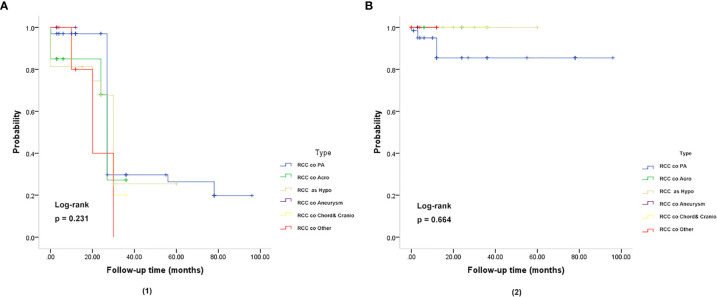
Kaplan-Meier plot for overall survival (OS) **(A)** and relapse-free survival (RFS) **(B)** in patients with RCC coexisting with various sellar lesions. (**A**, 1): Kaplan-Meier plot for overall survival in patients. The solid blue line represents patients with the coexistence of RCC and PA (n = 67). The solid green line represents patients with the coexistence of RCC and somatotroph adenoma (acromegaly) (n = 20). The solid yellow line represents patient with the coexistence of RCC and hypophysitis (n = 16). The solid purple line represents patients with the coexistence of RCC and aneurysm (n = 4). The solid yellow line represents patients with the coexistence of RCC and chordoma and craniopharyngioma (n = 5). The solid red line represents patients with the coexistence of RCC and other sellar lesions (n = 6) (epidermoid cyst, dermoid cyst, pituitary granulomatosis, salivary tumor, salivary gland remnants). There was no significant difference in OS and RFS between the six subgroups of patients (*P* = 0.231). (**B**, 2): Kaplan-Meier plot showing the relapse-free survival (RFS) of patients. There was no significant difference in OS and RFS among the six subgroups of patients (*P* = 0.664). The *P*-values were obtained by the log-rank (Mantel-Haenszel) test.

In the follow-up analysis, various subgroups showed no difference in overall survival (OS), and relapse-free survival (RFS) (*P* = 0.231, *P* = 0.664) (**Figure 3**). The Median overall survival period was 22.93 months. The 3- and 5-year OS rates were 95.50 and 95.77%, respectively, which did not differ based on imaging evidence. In this group, 2 (2.98%) patients experienced recurrence.

### Risk of Bias (Methodological Quality)

**Figure 4:**
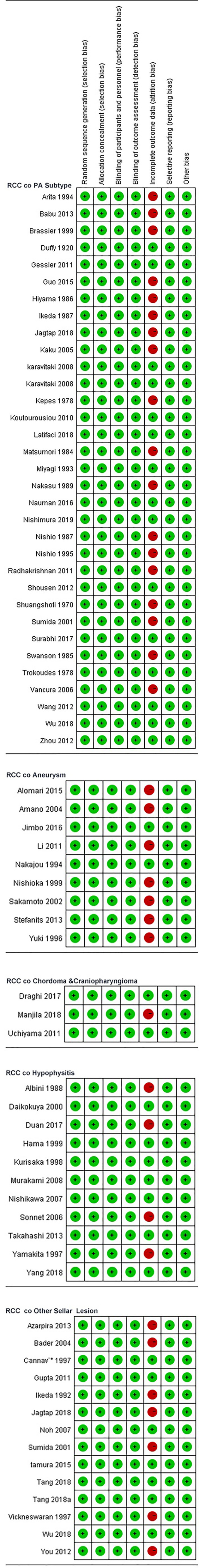
Review author's judgements about each risk of bias item for each included case series /case-control.

The risk of bias in the included case series/case-control original studies is shown in **Figure 4**. The bias risk of all studies in the literature was evaluated in seven domains; 50 studies (case series) and 17 studies (case controls) were at risk of unbiased data (attrition bias) and were considered low risk in terms of bias. In terms of bias in measuring the results, only one study in the case series had incomplete outcome data (attrition bias).

### Disease Model Validation

A 23-year-old Chinese man presented to the clinic 2 days following sudden onset of headache and vomiting. Neurological examination revealed no focal deficit include oculomotor palsy or visual field defect. Computerized tomography (CT) and MRI image showed 2.5 × 2.4 × 2.3 cm cystic-solid lesion in the sellar and suprasellar regions, which had low signal intensity on T1-weight image and high signal intensity on T2-weighted image. The cystic mass located between the anterior and posterior pituitary lobes, and no evidence of SAH (**Case illustrate - Figure 1A**). A disease was suspected. Endocrinological evaluation revealed a serum prolactin level of 12.0 ng/mL (reference range, normal 2.1–11.7 ng/mL), testosterone level of 255.9 ng/dL (reference range, normal 358–1217 pg/dL), and estradiol level of 0.0 pg/mL (reference range, normal 19.9–47.9 pg/dL), adrenocorticotrophic hormone, follicle stimulating hormone, luteinizing hormone, growth hormone, insulin-like growth factor, thyroid stimulating hormone, free T3, free T4, and cortisol were within normal limits.

The patient underwent microsurgical resection using the trans-sphenoidal surgery (TSS) approach under general anesthesia. Intraoperatively, the cyst was found to contain whitish yellow free-flowing mucus (**Case illustrate - Figure 1**) the pathology was consistent with that of an RCC (**Case illustrate - Figure 3**). Gross total resection (complete cyst and wall excision) was performed without intraoperative tearing of the arachnoid membrane or subsequent cerebrospinal fluid (CSF) leakage and massive hemorrhage.

The patient’s level of arousal at postoperative 24 hours decreased to somnolence. The pupils were equal and reactive to light bilaterally. The visual fields were full, and the extraocular eye movements were intact. Laboratory test results were normal. Postoperative CT (postoperative day 1) revealed Fisher grade IV SAH (**Case illustrate - Figure 1**). Computerized tomographic angiography (CTA) demonstrated a 5 mm AComA aneurysm with diffuse perfusion delay in the anterior longitudinal fissure and bilateral sylvian fissures (**Case illustrate - Figure 2**). The patient underwent a right frontotemporal craniotomy to treat the aneurysm (postoperative day 3). When the dura mater was opened, the brain was found to be swollen and the SAH was clearly observed. Intraoperative findings were consistent with that of a ruptured AComA aneurysm (**Case illustrate - Figure 2**). The patient recovered well following craniotomy and clipping of the aneurysm and was discharged home 5 days following the clipping. There was no symptomatic cerebral vasospasm or hydrocephalus. At 6 months follow-up, visual field acuity had fully recovered to the preoperative level, and physical examination findings remained unchanged. A repeat CT and MRI of the brain with gadolinium showed no residual tumor or cyst recurrence.

## Discussion

S-ErACT is a rare entity that consists of two distinct neoplasms that have an embryological ancestry (or share common embryologic ancestry), develop in juxtaposition to one another and have no or varying degrees of involvement intermingling between them; moreover, one of the tumors may exhibit characteristics of pluripotent stem cells (stratified squamous epithelial progenitor cells) (7). The use of preoperative MRI/CT diagnosis to distinguish both lesions is challenging, as they share similarities in radiological appearance and are often incidental. However, the presence of both lesions significantly alters the biological behavior of tumors and can be mistaken for cystic sellar lesions (17, 18), and postoperative pathological diagnosis is the only main basis for distinguishing the two.

Whereas the results of analyses on the outcomes of RCC coexisting with sellar lesions in adults are available, there are no such studies on different age groups and the clinicopathology of sellar collision lesions. Thus far, it is known from the MEDLINE database that embryonic remnants are significant risk factors for sellar tumors. In this study, the incidence rates of concomitant sellar lesions were influenced by the presence of RCC, PA, age, and S-ErACTs but not by location. The risk of coexistence was most significant in patients aged 30–39 years (PA), 50–59 years (SA), 12–19 years (Hypo), and ≥60 years (aneurysm), compared with those who were in other age groups (various sellar lesions). Women had a higher prevalence of the coexistence of RCC with sellar lesions than men. Patients with coexisting PA and Hypo or CD and CP had a higher prevalence than those with coexisting aneurysm. The coexistence of RCCs and AComA aneurysms has rarely been reported, and systematic reviews of just four cases (including our one case) have been published from 1920 to 2018 (19–22).

We calculated that in a population of people with collision lesions consisting of men and females of various ages, the incidence rates of RCC coexisting with PA ranged from 0.02 to 57.91%. This is higher than that in previous studies, which reported a range of 1.7–1.9%. This difference can be explained by adjustments for age and sex in retrospective studies. Our present findings are probably more accurate because we included approximately several times more studies than previous works, and more importantly, we were able to adjust IR for different age groups.

Different age groups and histopathological characteristics are major risk factors for RCC coexisting with various sellar lesions, and patients who have sellar embryonic remnants are at increased risk of developing this disease over time. Owing to abundant data, we could separately assess the incidence of concomitance (RCC and various sellar lesions) in different age groups and patients with various subtypes of coexistence of sellar lesions. For patients who are in different age groups and have sellar embryonic remnant-associated disease, there may be a clear association. This means that these embryonic residues, which should have disappeared after birth or with development, are affected by acquired factors, such as hormone axes and changes in the developmental environment.

**Figure 5:**
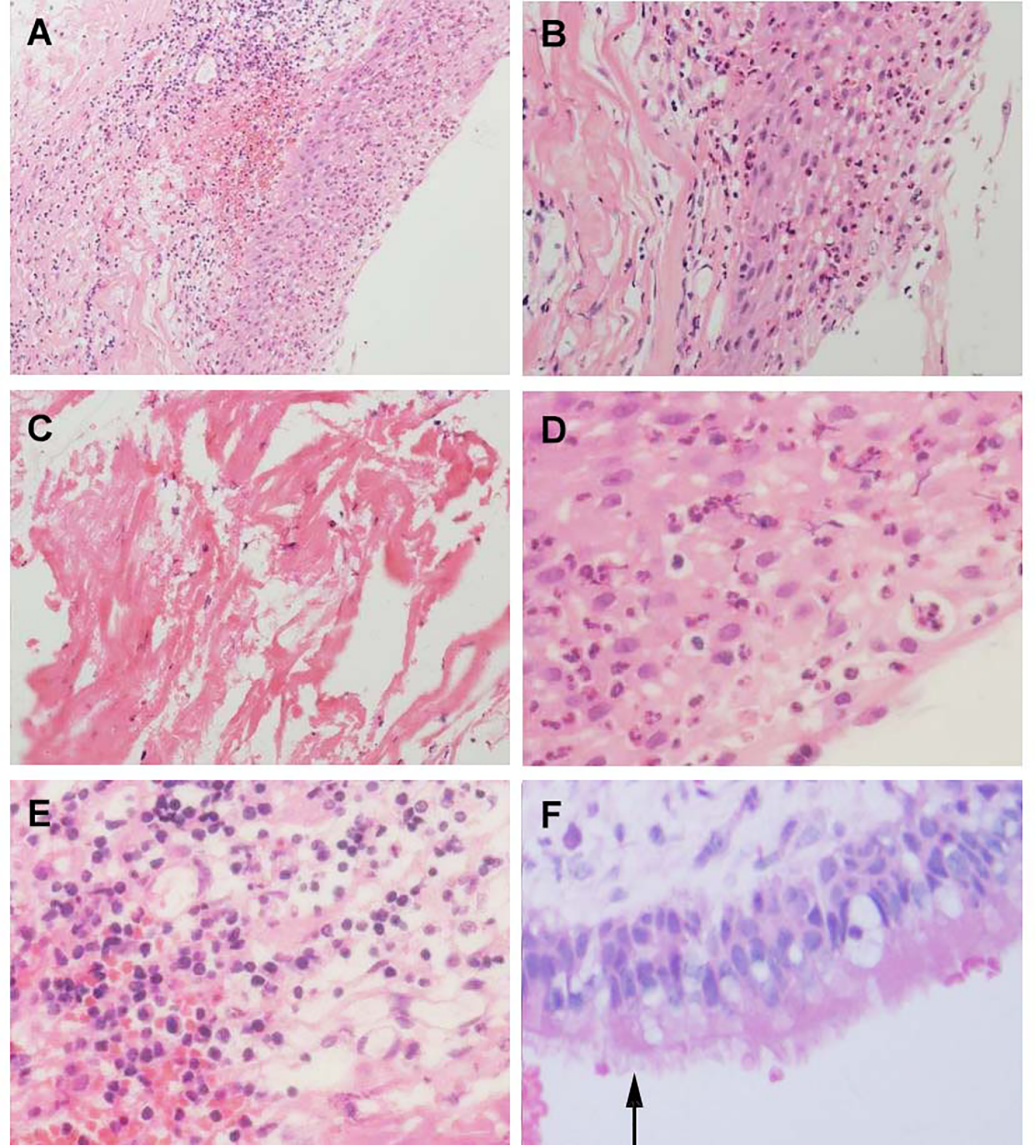
Histological feature of Aneurysm with Rathke’s cleft cyst, H&E stained section. **(A)** Shows the histological features of simple partial covered squamous epithelium with fibrous connective tissue presents acute and chronic inflammation (×150). **(B)** Squamous epithelial mucosa (×300). **(C)** The keratin in the cyst wall. **(D)** cystic wall lined by a squamous epithelium (×400). **(E)** Groups of inflammation cells (400×). **(F)** cuboidal ciliated epithelium cells(arrows) (×400).

**Figure 6:**
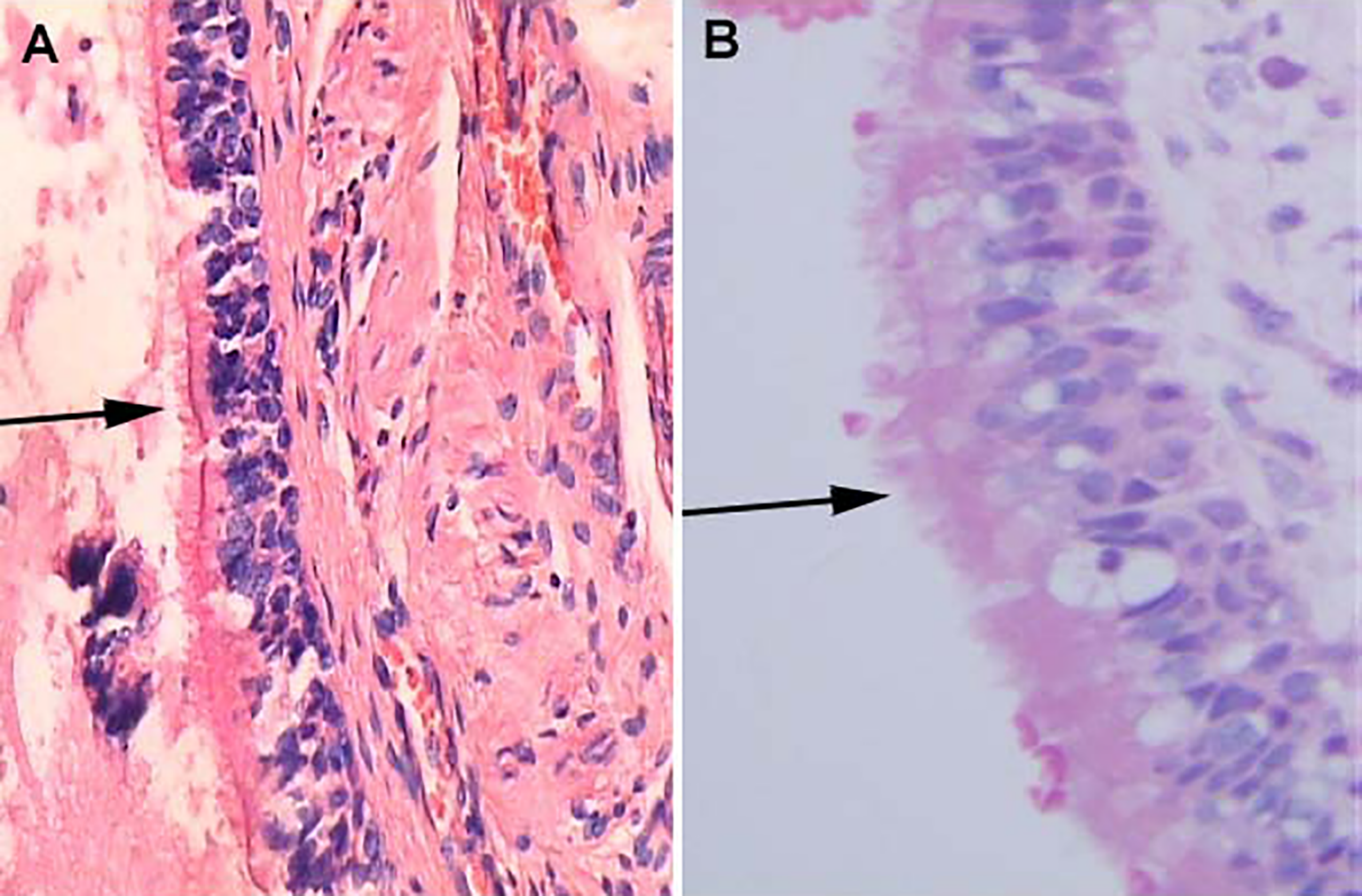
Histological features of Human bronchial epithelium and Rathke’s cleft cyst epithelium, H&E stained section. **(A)** Ciliated bronchial epithelium (arrows) (×100). **(B)** Cuboidal ciliated epithelium (arrows) (×400).

Although collision lesions of the sellar region are incidentally found at the clinic or on imaging, the rate of asymptomatic cystic lesions identified at autopsy is as high as 33% (9). The cell distribution of the various embryonic remnants in histopathological patterns is as follows: ciliated columnar cells or monolayers of cuboidal, stratified squamous epithelium, inflammatory cells, respiratory type epithelium, cilia, and mucous secreting goblet cells, and some were squamous metaplasia (see **Figure 5**). They have a similar location in the sellar and suprasellar regions and show overlapping histopathologic features, namely, ciliated columnar epithelium to ciliated squamous epithelium to squamous epithelium. This reflects the process of lesion development; the changes in these pathological cells are similar to embryonic esophageal epithelial replacement during the embryonic period. These pathological changes in the respiratory-type epithelium (see **Table 3**) prompted us to examine the bronchial epithelial structure. In contrast, the pathological structure of the RCC cyst wall was surprisingly similar to that of bronchial epithelial cells (see **Figure 6**), suggesting that the presence of squamous metaplasia or pseudostratified cuboidal cells in the cyst wall was regenerative and revealing that this layer of cells has the characteristics of progenitor cells or stem cells. Recent experimental studies have also confirmed this view (23). Another study suggested that squamous metaplasia and isointensity on T2-weighted MRI were independent predictors of cyst recurrence (24).

The changes in RCC pathological cells and the similarity between the pathological structures and the bronchial epithelium structure support the theory of the development of the pharyngeal bursa after migration (25). There is compelling evidence to show that a proportion of stem cells around the pituitary cleft and marginal zone in the pituitary or epithelial cells in Rathke’s pouch wall formed by mesenchyme accumulation may undergo further differentiation in response to some growth (23, 26–28). Under different inducing factors, these progenitor cells behave similarly to the transition cells proposed by Kepes (29). This finding illustrates the replacement/or renewal process to transitional and accumulation of epithelial cells (30). In a more recent report published in 2018, four cases of previously diagnosed RCC were reclassified as papillary craniopharyngioma because they were BRAF^V600E^ positive, implying the transformation of RCC into papillary craniopharyngioma (16).

This evidence supports our study as the basis for the theory of the postmigration development of the pharyngeal bursa. However, evidence of a common origin remains controversial. To our knowledge, PA is the independent risk factor with the highest risk (minimal difference in collision accuracy) according to the pathological graded risk for association with RCC. Interestingly, we found that gonadotroph hormone adenoma (HR 2.719) and corticotrophin hormone adenoma (HR 2.652) had the highest risk of merging with RCC among the pathological subtypes of PA (**Table 2**). Evaluations of both age and pathology for sellar lesions may provide meaningful information to determine whether there are pathological structures and cells with potential stem cell characteristics in constantly changing pathological tissues and concomitant/associated diseases in the sellar region.

In fact, we observed differentiated epithelial cells in RCC, from single columnar epithelium to ciliated epithelium, and GC (HR 13.195, P = 0.005) and/or stratified epithelium had a significantly higher risk of replacement/renewal to transitional/accumulation. This finding shows the process of replacement/renewal or transition and accumulation of epithelial cells, which may arise from Rathke’s pouch parietal cells, as they are composed of progenitor cells, which directly differentiate from stem cells. These lesions (RCC co PA) have been found to contain ciliated columnar cells or monolayers of cuboidal cells and show different incidence rates (IR) ranging from 0.02 to 27.62%, suggesting an intermediate entity between RCC and sellar lesions, and this evidence lends support to this idea (13).

Our data also demonstrated that the different sellar embryonic remnants associated with tumor progression represent a multistep process that involves age and the accumulation of heterogeneous genetic mutations. Previous studies have reported that the pathological findings of various diseases in the sellar region are consistent with RCC embryology and elements (6, 31). However, it has recently been asserted that the persistence of the embryonal morphology of the infundibular recess might then be the result of dysembryogenesis in humans. No data have yet confirmed the neoplastic nature of the lesion (8). In addition, evaluating RCCs and sellar lesions is useful because it yields information on RCCs and the pathological tissue in sellar lesions. However, its role in pathogenesis remains unclear.

This study has several limitations. First, it was a retrospective study, so unknown biases could be present, and any biases would affect positive results. Another limitation is the small sample size. We only provide pooled data for patients who had reported morbidities, but those who were already sick and reported were not included (patients not included because of incomplete full-text retrieval or inconsistent data). Thus, more studies are needed to focus on such patients. The unknown heterogeneity of the included studies in terms of study design, regions of study, time of onset, age at diagnosis, living environment, and other factors made it difficult to achieve valid and stable meta-analysis.

## Conclusions

These pathological changes represent crucial information on sellar embryonic remnant-associated collision tumors and provide necessary clinical observation data to these tumors. Exploratory traceability studies have shown that our clinical observation data verify the accumulation process from ciliated columnar epithelium to ciliated squamous epithelium to squamous epithelium and that the RCC cyst wall has tracheobronchial airway epithelium with similar characteristics to progenitor cells. It is necessary to further expand this work to find more evidence.

## Data Availability Statement

The datasets presented in this study can be found in online repositories. The names of the repository/repositories and accession number(s) can be found in the article/**Supplementary Material**.

## Ethics Statement

The study was approved by the ethics committee in Peking Union Medical College Hospital, China. Written informed consent was obtained from the legal representatives of all participants before inclusion (approval date. 2011-0831).

## Author Contributions

Conception and design: MW and WM. Acquisition of data: MW, ZW, ZZ, MS, and WM. Analysis and interpretation of data: MW and MS. Drafting the article: MW, WM, and ZZ. Critically revising the article: ZW, WM, and ZZ. Reviewed submitted version of manuscript: all authors. Approved the final version of the manuscript on behalf of all authors: WM and ZW. Statistical analysis: MW, MS, and QF. Administrative/technical/material support: ZZ, ZW, JZ, RW, and WM. Study supervision: ZW, JZ, RW, WM, and ZZ. All authors contributed to the article and approved the submitted version.

## Funding

This research did not receive any specific grant from funding agencies in the public, commercial, or not-for-profit sectors.

## Conflict of Interest

The authors declare that the research was conducted in the absence of any commercial or financial relationships that could be construed as a potential conflict of interest.

## Abbreviations

Acro, acromegaly; AComA, anterior communicating artery; AUC, areas under curves; AT/RT, atypical teratoid rhabdoid tumor; CA-ccc, ciliated columnar cell or monolayer of cuboidal; CP-ICA, cavernous portion of the right internal carotid artery; CD, chordoma; CP, craniopharyngioma; CT, computerized tomography; CTA, computerized tomographic angiography; CI, confidence interval; CTs, collision tumors; DC, dermoid cyst; DI, diabetes insipidus; DSA, digital subtraction angiography; EC, epidermoid cyst; EC-CC, epithelial cells with ciliated cuboidal; EM, epithelial cells and multinucleated giant cells or cholesterin; FM, foamy macrophages; KM, Kaplan-Meier; Hypo, hypophysitis, HR, hazard ratio; IR, incidence rates; GA, gonadotroph adenomas; GC, goblet cells; MRI, magnetic resonance imaging; MRA, magnetic resonance angiography; NFPA, non-functioning pituitary adenomas; OS, overall survival; PA, pituitary adenomas; PEIR, persisting embryonal infundibular recess; PIA, plurihormonal and double adenomas; PitNET, pituitary neuroendocrine tumor; PG, pituitary granulomatosis; ROC, receiver operating characteristic; RCTs, randomized controlled trials; RCCs, Rathke’s cleft cysts; RFS, relapse free survival; SA, somatotroph adenoma; S-ErCT, sellar embryonic-remnants associated collision tumors; SGR, salivary gland remnants; SM, squamous metaplasia; SSEC, stratified squamous epithelium cell; TSS, trans-sphenoidal surgery; TCT, transitional cell tumor.
