# Supplementary material for: A Potential Concomitant Sellar Embryonic Remnant-Associated Collision Tumor: Systematic Review
Source: Front Oncol. 2021 Apr 29;11:649958. doi: 10.3389/fonc.2021.649958 (PMC8117962; doi:10.3389/fonc.2021.649958)
Supplement: Supplementary file 1 [file DataSheet_1.docx]

**Case illustrate Supplemental Files**------- **A Potential Concomitant Sellar lesion and Embryonic- Remnants associated Tumor**

Mingdong Wang^1^; Qianhui Fu^2^; Mingjing Song^3^;Zongmao Zhao^4^; Renzhi Wang^5^; John Zhang^6^; Wenbin Ma **^5^**; Zhanxiang Wang^1^

**Available data (Case illustrate)---**A 23-year-old Chinese man presented to the clinic 2 days following sudden onset of headache and vomiting. Neurological examination revealed no focal deficit include oculomotor palsy or visual field defect. Computerized tomography (CT) and MRI image showed 2.5 x 2.4 x 2.3 cm cystic-solid lesion in the sellar and suprasellar regions, which had low signal intensity on T_1_-weight image and high signal intensity on T_2_-weighted image. The cystic mass located between the anterior and posterior pituitary lobes, and no evidence of SAH (**Case illustrate** -Fig. 1A). A disease was suspected. Endocrinological evaluation revealed a serum prolactin level of 12.0 ng/mL (reference range, normal 2.1–11.7 ng/mL), testosterone level of 255.9 ng/dL (reference range, normal 358–1217 pg/dL), and estradiol level of 0.0 pg/mL (reference range, normal 19.9–47.9 pg/dL)，adrenocorticotrophic hormone, follicle stimulating hormone, luteinizing hormone, growth hormone, insulin-like growth factor, thyroid stimulating hormone, free T3, free T4, and cortisol were within normal limits.

The patient underwent microsurgical resection using the trans-sphenoidal surgery (TSS) approach under general anesthesia. Intraoperatively, the cyst was found to contain whitish yellow free-flowing mucus (**Case illustrate** -Fig.1) (suggesting a Rathke’s cleft cyst) and the pathology was consistent with that of an RCC (**Case illustrate** -Fig.3). Gross total resection (complete cyst and wall excision) was performed without intraoperative tearing of the arachnoid membrane or subsequent cerebrospinal fluid (CSF) leakage and massive hemorrhage.

The patient’s level of arousal at postoperative 24 hours decreased to somnolence. The pupils were equal and reactive to light bilaterally. The visual fields were full, and the extraocular eye movements were intact. Laboratory test results were normal. Postoperative CT (postoperative day 1) revealed Fisher grade IV SAH (**Case illustrate**-Fig.1). Computerized tomographic angiography (CTA) demonstrated a 5 mm AComA aneurysm with diffuse perfusion delay in the anterior longitudinal fissure and bilateral sylvian fissures (**Case illustrate-** Fig. 2). The patient underwent a right frontotemporal craniotomy to treat the aneurysm (postoperative day 3). When the dura mater was opened, the brain was found to be swollen and the SAH was clearly observed. Intraoperative findings were consistent with that of a ruptured AComA aneurysm (**Case illustrate**-Fig. 2). The patient recovered well following craniotomy and clipping of the aneurysm and was discharged home 5 days following the clipping. There was no symptomatic cerebral vasospasm or hydrocephalus. At 6 months follow-up, visual field acuity had fully recovered to the preoperative level, and physical examination findings remained unchanged. A repeat CT and MRI of the brain with gadolinium showed no residual tumor or cyst recurrence.
