# Supplementary material for: A Potential Concomitant Sellar Embryonic Remnant-Associated Collision Tumor: Systematic Review
Source: Front Oncol. 2021 Apr 29;11:649958. doi: 10.3389/fonc.2021.649958 (PMC8117962; doi:10.3389/fonc.2021.649958)
Supplement: Supplementary file 2 [file DataSheet_2.docx]

**Case illustrate** -Figure1 Imaging feature of RCCs. Coronal and Sagittal T1weighted MRI images showing (A/B) A Rathke’s cleft cyst with T1 isointense cyst contents, suggestive of low protein cyst content, as seen on inferior-contrast (A) and (B) post-contrast images with the contrast causing the anterior lobe of the gland to brighten. (C) Typical of aneurysms SAH pattern of haemorrhage, thick accumulation of blood in front of midbrain and diffuse at sylvan fissure, anterior longitudinal fissure and tentorium of cerebellum. (D) intraoperative the white-yellow mucus flowed from the cyst .





**Case illustrate** -Figure2 pre-operative computed tomography of angiography (CTA) (A/B) shows a right anterior cerebral artery aneurysm. Intra-operative visualization of features (C) Postoperative CT scan demonstrated satisfactory result. (D)An intraoperative photograph revealed the anterior cerebral artery after aneurysm clipping


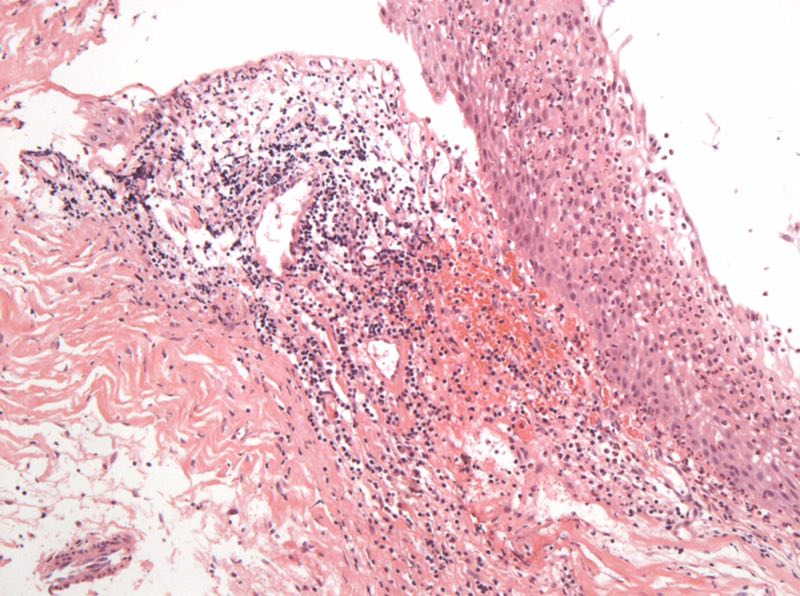


**Case illustrate** -Figure3 Hematoxylin-eosin staining. Shows the histological features of simple

partial covered squamous epithelium with fibrous connective tissue presents acute and chronic inflammation.
