# Supplementary material for: A Potential Concomitant Sellar Embryonic Remnant-Associated Collision Tumor: Systematic Review
Source: Front Oncol. 2021 Apr 29;11:649958. doi: 10.3389/fonc.2021.649958 (PMC8117962; doi:10.3389/fonc.2021.649958)
Supplement: Supplementary file 3 [file DataSheet_3.docx]

2021-04-12

**A Potential Concomitant Sellar Embryonic- Remnant associated Collision Tumor**

*Wang, et al*

Supplementary appendix-1

**Sections page**

**1) Systematic reviews and meta-analyses (PRISMA) guidelines and checklist 1**

**2) Inclusion criteria -** **The diagnosis standard of pituitary adenoma; Explanation of cases without modern imaging in the 1920s and 1970s Additional explanation 4**

**3) Statistical analysis 5-6**

**4) Divide into groups additional notes 7**

**5) Some problems that need further explanation 9**

**6) Identified sixty-seven meta-analyses 10**

**Supplementary Table 1: Checklist of study selection in the study**

| **Section/topic** | **#** | **Checklist item** | **Reported on page #** |
| --- | --- | --- | --- |
| **TITLE** | | |  |
| Title | 1 | Identify the report as a systematic review, meta-analysis, or both. | Page1 |
| **ABSTRACT** | | |  |
| Structured summary | 2 | Provide a structured summary including, as applicable: background; objectives; data sources; study eligibility criteria, participants, and interventions; study appraisal and synthesis methods; results; limitations; conclusions and implications of key findings; systematic review registration number. | Page2 |
| **INTRODUCTION** | | |  |
| Rationale | 3 | Describe the rationale for the review in the context of what is already known. | Page4 |
| Objectives | 4 | Provide an explicit statement of questions being addressed with reference to participants, interventions, comparisons, outcomes, and study design (PICOS). | Page6 |
| **METHODS** | | |  |
| Protocol and registration | 5 | Indicate if a review protocol exists, if and where it can be accessed (e.g., Web address), and, if available, provide registration information including registration number. | Page6 |
| Eligibility criteria | 6 | Specify study characteristics (e.g., PICOS, length of follow-up) and report characteristics (e.g., years considered, language, publication status) used as criteria for eligibility, giving rationale. | Page6 |
| Information sources | 7 | Describe all information sources (e.g., databases with dates of coverage, contact with study authors to identify additional studies) in the search and date last searched. | Page6 |
| Search | 8 | Present full electronic search strategy for at least one database, including any limits used, such that it could be repeated. | Page6 |
| Study selection | 9 | State the process for selecting studies (i.e., screening, eligibility, included in systematic review, and, if applicable, included in the meta-analysis). | Page7 |
| Data collection process | 10 | Describe method of data extraction from reports (e.g., piloted forms, independently, in duplicate) and any processes for obtaining and confirming data from investigators. | Page7 |
| Data items | 11 | List and define all variables for which data were sought (e.g., PICOS, funding sources) and any assumptions and simplifications made. | Page7 |
| Risk of bias in individual studies | 12 | Describe methods used for assessing risk of bias of individual studies (including specification of whether this was done at the study or outcome level), and how this information is to be used in any data synthesis. | Page8 |
| Summary measures | 13 | State the principal summary measures (e.g., risk ratio, difference in means). | Page8 |
| Synthesis of results | 14 | Describe the methods of handling data and combining results of studies, if done, including measures of consistency (e.g., I^2^) for each meta-analysis. | Page8 |

| **Section/topic** | **#** | **Checklist item** | **Reported on page #** |
| --- | --- | --- | --- |
| Risk of bias across studies | 15 | Specify any assessment of risk of bias that may affect the cumulative evidence (e.g., publication bias, selective reporting within studies). | Page8 |
| Additional analyses | 16 | Describe methods of additional analyses (e.g., sensitivity or subgroup analyses, meta-regression), if done, indicating which were pre-specified. | Page8 |
| **RESULTS** | | |  |
| Study selection | 17 | Give numbers of studies screened, assessed for eligibility, and included in the review, with reasons for exclusions at each stage, ideally with a flow diagram. | Page8 |
| Study characteristics | 18 | For each study, present characteristics for which data were extracted (e.g., study size, PICOS, follow-up period) and provide the citations. | Page9 |
| Risk of bias within studies | 19 | Present data on risk of bias of each study and, if available, any outcome level assessment (see item 12). | Page9 |
| Results of individual studies | 20 | For all outcomes considered (benefits or harms), present, for each study: (a) simple summary data for each intervention group (b) effect estimates and confidence intervals, ideally with a forest plot. | Page10 |
| Synthesis of results | 21 | Present results of each meta-analysis done, including confidence intervals and measures of consistency. | Page11 |
| Risk of bias across studies | 22 | Present results of any assessment of risk of bias across studies (see Item 15). | Page12 |
| Additional analysis | 23 | Give results of additional analyses, if done (e.g., sensitivity or subgroup analyses, meta-regression [see Item 16]). | Page12 |
| **DISCUSSION** | | |  |
| Summary of evidence | 24 | Summarize the main findings including the strength of evidence for each main outcome; consider their relevance to key groups (e.g., healthcare providers, users, and policy makers). | Page13 |
| Limitations | 25 | Discuss limitations at study and outcome level (e.g., risk of bias), and at review-level (e.g., incomplete retrieval of identified research, reporting bias). | Page16 |
| Conclusions | 26 | Provide a general interpretation of the results in the context of other evidence, and implications for future research. | Page17 |
| **FUNDING** | | |  |
| Funding | 27 | Describe sources of funding for the systematic review and other support (e.g., supply of data); role of funders for the systematic review. | Page18 |

**The diagnosis standard of pituitary adenoma**

The diagnosis criteria for pituitary adenoma is based on the classification of classification of tumors of the pituitary gland by WHO in 2017 (1).

**Inclusion criteria-- Explanation of cases without modern imaging in the 1920s and 1970s Additional explanation**

CT, MRI, DSA, CTA, and MRA were certainly not available until the early 70ies. Prior to the use neuroimaging, light microscope and clinical histopathologic examinations have been used for studies in clinical practice. Based to the histopathological performance, combined with the clinical endocrine characteristics and clinical manifestations, we meet the diagnosis criteria of 2017.

**Statistical methods**

We first used R programming language version 3.4 (statistic package studio) to implement Random forest algorithm and get the important Categorical variable score (We identified sixty-seven meta-analyses (30-94) ^30-94^. Test for association between unitary-factor and the clinical surgery was the outcome. A series of correlation analysis were preformed to observe the age, histopathology trend of the data and verify the presence of significant Risk of merge of two diseases, developmental relationship between age and RCC coexisting sellar lesion risk in the outcomes. The statistical heterogeneity between specific OR by the *I^2^* statistic test and Quantitative test, specificity yielded a *P* value.

Next, we performed several post-hoc sensitivity analysis. For comparisons between different age groups, we conducted multiple comparison into account by using Parirwise comparison as a post–hoc test. We illustrated differ age subgroup sensitivity and specificity through curves of the receiver operating characteristic (AUC). The Cox proportional hazard model was used for hazard ratios (HR) with 95% confidence intervals (CIs) were used to quantify the strength of the association between risk coexistent and age. That evaluated whether age was a significant direct effect, between variable, age and RCC coexisting sellar lesion in the present cases, risk of merger of two disease. Levene’s Test was used to test the homogeneity of variance prior to ANOVA analysis, and measured age and RCC coexistent sellar lesion inter-observer variability.

Finally, we conducted two multivariate logistic regression analysis to identify the predictors of two disease coexistent, various subtype pathological (HR and sensitivity, specificity) and the predictively accuracy. A Cox proportional-hazard model for multivariable analysis was applied for variables (subtype pituitary adenoma, other sellar region lesions) that proved to be significant in the univariate. Hazard ratios (HR) with 95% confidence interval (95% CI) were determined using univariate or multivariable analysis. We used the Cox hazard regression model to calculate the risk degree of pathology typing. Receiver operating characteristic (ROC) analysis was used to identify the most disease likely to merge (various types of PA, Hypo, aneurysm, Chor & CP, and other sellar lesion) by plotting evaluation by sellar region lesion coexistence RCC status, and predictive accuracy was determined by measuring area under the ROC curve-AUC, specificity and sensitivity. AUC of 0.7 was considered to be sufficiently discriminative; AUC of 0.5 is equivalent to a coin toss. The most suitable univariate was selected by comparing the area under the curves (AUC) from the ROC curves for Cut point and by performing logistic regression analysis.

The Kaplan-Meier (KM) method overall survival (OS) and relapse free survival (RFS) analysis was performed for six subgroups of patients. Classified on the basis of coexisting with various sellar lesion. The significant of the survival curve was assessed using the log-rank (Mantel- Haenszel) test.

**Divide into groups additional notes**

In the retrieved literature, we can see that the GH adenoma (acromegaly,Hematoxylin and eosin-H&E, immunohistochemistry-IHC) is the most common type. What causes this phenomenon? Base on this point view, we subdivided the pituitary adenoma group to separate the Somatotroph (acromegaly) adenoma.

**Clinically nonfunctional /silent adenoma -Some problems that need further explanation**

We classify according to the 2017 WHO classification of tumors of the pituitary gland, which is evolved from that based on the expression of adeno-hypophyseal hormones and immunohistochemical hormone expression and ultrastructural characteristics method. Hormone-negative adenoma are not equivalent to nonfunctioning adenoma, as IHC can be expressed in several adenoma types yet be clinically silent. The majority of nonfunctional adenoma literature are found to be gonadotroph adenomas and null cell tumors nearly half of nonfunctioning tumors. A significant subset of hormone-negative adenomas included silent Corticotroph adenoma. Silent ACTH adenoma, clinically silent GH PRL, and TSH adenoma are rare. Patients with nonfunctioning adenomas and acromegaly have also been shown to have a significantly higher inci­dence of malignancy than the general population (5). only around 2% of non-functioning pituitary neuroendocrine tumors lack signs of pituitary cell lineage differentiation, being thus classified as null-cell adenomas (1,2,3,4).

**Why combining chordoma and cranipopharyngioma?**

Despite they are not even remotely associated with each other (diagnostically). However, In terms of origin, embryogenic the notochordal originates from occipital bone, the notochord induces the outpouching of the roof of the stomodeal ectoderm that results in formation of Rathke’s pouch. Base on this point view, we put two disease in one subgroup

Reference

1.M. Beatriz S. Lopes.The 2017 World Health Organization classification of tumors of the pituitary gland: a summary. Acta Neuropathol. 2017;134: 521–535

2. DeLellis RA. Pathology and genetics of tumours of endo- crine organs. World Health Organization classification of tumours, vol 8. International Agency for Research on Cancer, Lyon. 2004.

3. Grossman AB. The 2004 World Health Organization classification of pituitary tumors: is it clinically helpful? Acta Neuro- pathol. 2006; 111(1):76–77

4. Lloyd RV, Osamura RY, Kloppel G, Rosai J .2017. WHO classification of tumours of endocirne organs, 4th edn. International Agency for Research on Cancer, Lyon

5.Popovic V, Damianovic S, Micic D. et al. Increased incidence of neoplasia in patients with pituitary adenoma. Clin Endocrinol.1998,49;441-445.

**Identified sixty-seven literature meta-analyses**

1. [Azarpira N](https://www.ncbi.nlm.nih.gov/pubmed/?term=Azarpira%20N%5BAuthor%5D&cauthor=true&cauthor_uid=24101276), [Pakbaz S](https://www.ncbi.nlm.nih.gov/pubmed/?term=Pakbaz%20S%5BAuthor%5D&cauthor=true&cauthor_uid=24101276), [Torabineghad S](https://www.ncbi.nlm.nih.gov/pubmed/?term=Torabineghad%20S%5BAuthor%5D&cauthor=true&cauthor_uid=24101276), [Musavi J](https://www.ncbi.nlm.nih.gov/pubmed/?term=Musavi%20J%5BAuthor%5D&cauthor=true&cauthor_uid=24101276), [Rakei M](https://www.ncbi.nlm.nih.gov/pubmed/?term=Rakei%20M%5BAuthor%5D&cauthor=true&cauthor_uid=24101276). Acromegaly associated with mixed pituitary adenoma-gangliocytoma and Rathke 's cleft cyst. [*Turk Neurosurg.*](https://www.ncbi.nlm.nih.gov/pubmed/24101276) 2013;**23** (4): 527-30.
2. Arita K, Uozumi T, Takechi A, Hirohata T, Pant B, Kubo K, Tanaka K: A case of Cushing’s disease accompanied by Rathke’s cleft cyst: The usefulness of cavernous sinus sampling in the localization of microadenoma. Surg Neurol.1994; 42:112-116.
3. Geddes JF, Jansen GH, Robinson SF, Gömöri E, Holton JL, Monson JP, Besser GM, Révész T: ‘‘Gangliocytomas’’ of the pituitary. A heterogeneous group of lesion with differing histogenesis. *Am J Surg Pathol.* 2000; 24:607-613.
4. Miyagi A, Iwasaki M, Shibuya T, Kido G, Kushi H, Miyagami M, Tsubokawa T: Pituitary adenoma combined with Rathke’s cleft cyst-case report. *Neurol Med Chir (Tokyo)*.1993; 33:643-650.
5. Nishio S, Fujiwara S, Morioka T, Fukui M: Rathke’s cleft cysts within a growth hormone producing pituitary adenoma. *Br J Neurosurg* **.**1995; **9**(1):51-56.
6. Sumida M, Migita K, Tominaga A, Iida K, Kurisu K: Concomitant pituitary adenoma and Rathke’s cleft cyst. *Neuroradiology.* 2001; 43:755-759.
7. Duan K, Asa SL, Winer D, Gelareh Z, Gentili F, Mete O. [Xanthomatous Hypophysitis Is Associated with Ruptured Rathke's Cleft Cyst.](https://www.ncbi.nlm.nih.gov/pubmed/28120170) Endocr Pathol. 2017;**28**(1):83-90.
8. [Kaku S](https://www.ncbi.nlm.nih.gov/pubmed/?term=Kaku%20S%5BAuthor%5D&cauthor=true&cauthor_uid=16095210), [Tanaka T](https://www.ncbi.nlm.nih.gov/pubmed/?term=Tanaka%20T%5BAuthor%5D&cauthor=true&cauthor_uid=16095210), [Sawauchi S](https://www.ncbi.nlm.nih.gov/pubmed/?term=Sawauchi%20S%5BAuthor%5D&cauthor=true&cauthor_uid=16095210), [Dobashi H](https://www.ncbi.nlm.nih.gov/pubmed/?term=Dobashi%20H%5BAuthor%5D&cauthor=true&cauthor_uid=16095210), [Ohtsuka T](https://www.ncbi.nlm.nih.gov/pubmed/?term=Ohtsuka%20T%5BAuthor%5D&cauthor=true&cauthor_uid=16095210), [Numoto RT](https://www.ncbi.nlm.nih.gov/pubmed/?term=Numoto%20RT%5BAuthor%5D&cauthor=true&cauthor_uid=16095210), [Murakami S](https://www.ncbi.nlm.nih.gov/pubmed/?term=Murakami%20S%5BAuthor%5D&cauthor=true&cauthor_uid=16095210), [Komine K](https://www.ncbi.nlm.nih.gov/pubmed/?term=Komine%20K%5BAuthor%5D&cauthor=true&cauthor_uid=16095210), [Abe T](https://www.ncbi.nlm.nih.gov/pubmed/?term=Abe%20T%5BAuthor%5D&cauthor=true&cauthor_uid=16095210). A case of non-functional pituitary adenoma associated with Rathke's cleft cyst*.* [*No Shinkei Geka.*](https://www.ncbi.nlm.nih.gov/pubmed/16095210) 2005; **33**(8):797-803.
9. [Noh SJ](https://www.ncbi.nlm.nih.gov/pubmed/?term=Noh%20SJ%5BAuthor%5D&cauthor=true&cauthor_uid=17914599), [Ahn JY](https://www.ncbi.nlm.nih.gov/pubmed/?term=Ahn%20JY%5BAuthor%5D&cauthor=true&cauthor_uid=17914599), [Lee KS](https://www.ncbi.nlm.nih.gov/pubmed/?term=Lee%20KS%5BAuthor%5D&cauthor=true&cauthor_uid=17914599), [Kim SH](https://www.ncbi.nlm.nih.gov/pubmed/?term=Kim%20SH%5BAuthor%5D&cauthor=true&cauthor_uid=17914599). Pituitary adenoma and concomitant Rathke's cleft cyst. [*Acta Neurochir (Wien).*](https://www.ncbi.nlm.nih.gov/pubmed/17914599) 2007;**149**(12):1223-8.
10. Ikeda H, Yoshimoto T, Katakura R. A case of Rathke’s cleft cyst within a pituitary adenoma presenting with acromegaly-do ‘‘transitional cell tumours of the pituitary gland’’ really exist? *Acta Neuropathol (Berl*). 1992;83: 211–215.
11. Vancura RW, Jacob KM, Damjanov I . A 70-year-old man with diplopia, nausea, and vomiting. *Arch Pathol Lab Med.* 2006; 130: 403–404.
12. BaderLJ,CarterKD,LatchawRE,EllisWG,WexlerJA,WatsonJC. Simultaneous symptomatic Rathke’s cleft cyst and GH secreting pituitary adenoma: a case report. *Pituitary.* 2004; 7: 39–44.
13. [Uppal S](https://www.ncbi.nlm.nih.gov/pubmed/?term=Uppal%20S%5BAuthor%5D&cauthor=true&cauthor_uid=28500832), [Jee YH](https://www.ncbi.nlm.nih.gov/pubmed/?term=Jee%20YH%5BAuthor%5D&cauthor=true&cauthor_uid=28500832), [Lightbourne M](https://www.ncbi.nlm.nih.gov/pubmed/?term=Lightbourne%20M%5BAuthor%5D&cauthor=true&cauthor_uid=28500832), [Han JC](https://www.ncbi.nlm.nih.gov/pubmed/?term=Han%20JC%5BAuthor%5D&cauthor=true&cauthor_uid=28500832), [Stratakis CA](https://www.ncbi.nlm.nih.gov/pubmed/?term=Stratakis%20CA%5BAuthor%5D&cauthor=true&cauthor_uid=28500832). Combined pituitary hormone deficiency in a girl with 48, XXXX and Rathke's cleft cyst. [*Hormones* (Athens).](https://www.ncbi.nlm.nih.gov/pubmed/28500832) 2017;**16**(1):92-98.
14. [Stefanits H](https://www.ncbi.nlm.nih.gov/pubmed/?term=Stefanits%20H%5BAuthor%5D&cauthor=true&cauthor_uid=23254138), [Matula C](https://www.ncbi.nlm.nih.gov/pubmed/?term=Matula%20C%5BAuthor%5D&cauthor=true&cauthor_uid=23254138), [Frischer JM](https://www.ncbi.nlm.nih.gov/pubmed/?term=Frischer%20JM%5BAuthor%5D&cauthor=true&cauthor_uid=23254138), [Furtner J](https://www.ncbi.nlm.nih.gov/pubmed/?term=Furtner%20J%5BAuthor%5D&cauthor=true&cauthor_uid=23254138), [Hainfellner JA](https://www.ncbi.nlm.nih.gov/pubmed/?term=Hainfellner%20JA%5BAuthor%5D&cauthor=true&cauthor_uid=23254138), [Woehrer A](https://www.ncbi.nlm.nih.gov/pubmed/?term=Woehrer%20A%5BAuthor%5D&cauthor=true&cauthor_uid=23254138). Innervated ectopic salivary gland associated with Rathke's cleft cyst clinically mimicking pituitaryadenoma. [*Clin Neuropathol.*](https://www.ncbi.nlm.nih.gov/pubmed/23254138) 2013;**32**(3):171-5.
15. [Yamakita N](https://www.ncbi.nlm.nih.gov/pubmed/?term=Yamakita%20N%5BAuthor%5D&cauthor=true&cauthor_uid=9099592), [Ikeda T](https://www.ncbi.nlm.nih.gov/pubmed/?term=Ikeda%20T%5BAuthor%5D&cauthor=true&cauthor_uid=9099592), [Murai T](https://www.ncbi.nlm.nih.gov/pubmed/?term=Murai%20T%5BAuthor%5D&cauthor=true&cauthor_uid=9099592), [Kimura M](https://www.ncbi.nlm.nih.gov/pubmed/?term=Kimura%20M%5BAuthor%5D&cauthor=true&cauthor_uid=9099592), [Komaki T](https://www.ncbi.nlm.nih.gov/pubmed/?term=Komaki%20T%5BAuthor%5D&cauthor=true&cauthor_uid=9099592), [Miura K](https://www.ncbi.nlm.nih.gov/pubmed/?term=Miura%20K%5BAuthor%5D&cauthor=true&cauthor_uid=9099592), [Iwamura M](https://www.ncbi.nlm.nih.gov/pubmed/?term=Iwamura%20M%5BAuthor%5D&cauthor=true&cauthor_uid=9099592), [Hirata T](https://www.ncbi.nlm.nih.gov/pubmed/?term=Hirata%20T%5BAuthor%5D&cauthor=true&cauthor_uid=9099592), [Umezaki](https://www.ncbi.nlm.nih.gov/pubmed/?term=Umezaki%5BAuthor%5D&cauthor=true&cauthor_uid=9099592). Panhypopituitarism due to Rathke's cleft cyst associated with pituitary oncocytoma. [*Intern Med.*](https://www.ncbi.nlm.nih.gov/pubmed/9099592) 1997; **36**(2):107-12.
16. [Hiyama H](https://www.ncbi.nlm.nih.gov/pubmed/?term=Hiyama%20H%5BAuthor%5D&cauthor=true&cauthor_uid=3703147), [Kubo O](https://www.ncbi.nlm.nih.gov/pubmed/?term=Kubo%20O%5BAuthor%5D&cauthor=true&cauthor_uid=3703147), [Yato S](https://www.ncbi.nlm.nih.gov/pubmed/?term=Yato%20S%5BAuthor%5D&cauthor=true&cauthor_uid=3703147), [Kagawa M](https://www.ncbi.nlm.nih.gov/pubmed/?term=Kagawa%20M%5BAuthor%5D&cauthor=true&cauthor_uid=3703147), [Kitamura K](https://www.ncbi.nlm.nih.gov/pubmed/?term=Kitamura%20K%5BAuthor%5D&cauthor=true&cauthor_uid=3703147). A case of pituitary adenoma combined with Rathke's cleft cyst. [*No Shinkei Geka.*](https://www.ncbi.nlm.nih.gov/pubmed/3703147) 1986;**14**(3 Suppl):435-40.
17. [You C](https://www.ncbi.nlm.nih.gov/pubmed/?term=You%20C%5BAuthor%5D&cauthor=true&cauthor_uid=22824691), [Qiao F](https://www.ncbi.nlm.nih.gov/pubmed/?term=Qiao%20F%5BAuthor%5D&cauthor=true&cauthor_uid=22824691), [Jiang S](https://www.ncbi.nlm.nih.gov/pubmed/?term=Jiang%20S%5BAuthor%5D&cauthor=true&cauthor_uid=22824691), [Xiao A](https://www.ncbi.nlm.nih.gov/pubmed/?term=Xiao%20A%5BAuthor%5D&cauthor=true&cauthor_uid=22824691). Growth hormone secreting pituitary adenoma associated with Rathke's cleft cyst*.* [*Neurol India.*](https://www.ncbi.nlm.nih.gov/pubmed/22824691) 2012;**60**(3):310-1.
18. [Jimbo H](https://www.ncbi.nlm.nih.gov/pubmed/?term=Jimbo%20H%5BAuthor%5D&cauthor=true&cauthor_uid=26654927), [Ichikawa M](https://www.ncbi.nlm.nih.gov/pubmed/?term=Ichikawa%20M%5BAuthor%5D&cauthor=true&cauthor_uid=26654927), [Fukami S](https://www.ncbi.nlm.nih.gov/pubmed/?term=Fukami%20S%5BAuthor%5D&cauthor=true&cauthor_uid=26654927), [Otsuka K](https://www.ncbi.nlm.nih.gov/pubmed/?term=Otsuka%20K%5BAuthor%5D&cauthor=true&cauthor_uid=26654927), [Tsurukiri J](https://www.ncbi.nlm.nih.gov/pubmed/?term=Tsurukiri%20J%5BAuthor%5D&cauthor=true&cauthor_uid=26654927), [Sunaga S](https://www.ncbi.nlm.nih.gov/pubmed/?term=Sunaga%20S%5BAuthor%5D&cauthor=true&cauthor_uid=26654927), [Ikeda Y](https://www.ncbi.nlm.nih.gov/pubmed/?term=Ikeda%20Y%5BAuthor%5D&cauthor=true&cauthor_uid=26654927).Rapid De Novo Aneurysm Formation after Rathke Cleft Cyst Rupture*.* [*World Neurosurg.*](https://www.ncbi.nlm.nih.gov/pubmed/26654927) 2016;**88**:690.e11-6.
19. [Cannavò S](https://www.ncbi.nlm.nih.gov/pubmed/?term=Cannav%C3%B2%20S%5BAuthor%5D&cauthor=true&cauthor_uid=9125487), [Romano C](https://www.ncbi.nlm.nih.gov/pubmed/?term=Romano%20C%5BAuthor%5D&cauthor=true&cauthor_uid=9125487), [Buffa R](https://www.ncbi.nlm.nih.gov/pubmed/?term=Buffa%20R%5BAuthor%5D&cauthor=true&cauthor_uid=9125487), [Faglia G](https://www.ncbi.nlm.nih.gov/pubmed/?term=Faglia%20G%5BAuthor%5D&cauthor=true&cauthor_uid=9125487). Granulomatous sarcoidotic lesion of hypothalamic-pituitary region associated with Rathke's cleft cyst*.* [*J Endocrinol Invest.*](https://www.ncbi.nlm.nih.gov/pubmed/9125487) 1997; **20**(2):77-81.
20. [Murakami M](https://www.ncbi.nlm.nih.gov/pubmed/?term=Murakami%20M%5BAuthor%5D&cauthor=true&cauthor_uid=18521789), [Nishioka H](https://www.ncbi.nlm.nih.gov/pubmed/?term=Nishioka%20H%5BAuthor%5D&cauthor=true&cauthor_uid=18521789), [Izawa H](https://www.ncbi.nlm.nih.gov/pubmed/?term=Izawa%20H%5BAuthor%5D&cauthor=true&cauthor_uid=18521789), [Ikeda Y](https://www.ncbi.nlm.nih.gov/pubmed/?term=Ikeda%20Y%5BAuthor%5D&cauthor=true&cauthor_uid=18521789), [Haraoka J](https://www.ncbi.nlm.nih.gov/pubmed/?term=Haraoka%20J%5BAuthor%5D&cauthor=true&cauthor_uid=18521789). Granulomatous hypophysistis associated with rathke's cleft cyst: a case report. [*Minim Invasive Neurosurg.*](https://www.ncbi.nlm.nih.gov/pubmed/18521789) 2008;**51**(3):169-72.
21. [Koutourousiou M](https://www.ncbi.nlm.nih.gov/pubmed/?term=Koutourousiou%20M%5BAuthor%5D&cauthor=true&cauthor_uid=19551516), [Kontogeorgos G](https://www.ncbi.nlm.nih.gov/pubmed/?term=Kontogeorgos%20G%5BAuthor%5D&cauthor=true&cauthor_uid=19551516), [Wesseling P](https://www.ncbi.nlm.nih.gov/pubmed/?term=Wesseling%20P%5BAuthor%5D&cauthor=true&cauthor_uid=19551516), [Grotenhuis AJ](https://www.ncbi.nlm.nih.gov/pubmed/?term=Grotenhuis%20AJ%5BAuthor%5D&cauthor=true&cauthor_uid=19551516), [Seretis A](https://www.ncbi.nlm.nih.gov/pubmed/?term=Seretis%20A%5BAuthor%5D&cauthor=true&cauthor_uid=19551516). Collision sellar lesions: experience with eight cases and review of the literature. [*Pituitary.*](https://www.ncbi.nlm.nih.gov/pubmed/19551516) 2010;**13**(1):8-17.
22. [Nishikawa T](https://www.ncbi.nlm.nih.gov/pubmed/?term=Nishikawa%20T%5BAuthor%5D&cauthor=true&cauthor_uid=17384498), [Takahashi JA](https://www.ncbi.nlm.nih.gov/pubmed/?term=Takahashi%20JA%5BAuthor%5D&cauthor=true&cauthor_uid=17384498), [Shimatsu A](https://www.ncbi.nlm.nih.gov/pubmed/?term=Shimatsu%20A%5BAuthor%5D&cauthor=true&cauthor_uid=17384498), [Hashimoto N](https://www.ncbi.nlm.nih.gov/pubmed/?term=Hashimoto%20N%5BAuthor%5D&cauthor=true&cauthor_uid=17384498). Hypophysitis caused by Rathke's cleft cyst. Case report*.* [*Neurol Med Chir (Tokyo).*](https://www.ncbi.nlm.nih.gov/pubmed/17384498) 2007; **47**(3):136-9.
23. Kurisaka M, Fukui N, Sakamoto T, Mori K, Okada T, Sogabe K. [A case of Rathke's cleft cyst with apoplexy.](https://www.ncbi.nlm.nih.gov/pubmed/9726587) *Childs Nerv Syst*. 1998;**14**(7):343-7.
24. [Nakajou T](https://www.ncbi.nlm.nih.gov/pubmed/?term=Nakajou%20T%5BAuthor%5D&cauthor=true&cauthor_uid=8162156), [Morimoto M](https://www.ncbi.nlm.nih.gov/pubmed/?term=Morimoto%20M%5BAuthor%5D&cauthor=true&cauthor_uid=8162156), [Kurisaka M](https://www.ncbi.nlm.nih.gov/pubmed/?term=Kurisaka%20M%5BAuthor%5D&cauthor=true&cauthor_uid=8162156), [Mori K](https://www.ncbi.nlm.nih.gov/pubmed/?term=Mori%20K%5BAuthor%5D&cauthor=true&cauthor_uid=8162156). Two cases of craniopharyngioma associated with Rathke's cleft cyst*.* [*Noshuyo Byori.*](https://www.ncbi.nlm.nih.gov/pubmed/8162156) 1994;**11**(1):85-92.
25. [Yuki K](https://www.ncbi.nlm.nih.gov/pubmed/?term=Yuki%20K%5BAuthor%5D&cauthor=true&cauthor_uid=8672308), [Katsuzo K](https://www.ncbi.nlm.nih.gov/pubmed/?term=Katsuzo%20K%5BAuthor%5D&cauthor=true&cauthor_uid=8672308), [Ikawa F](https://www.ncbi.nlm.nih.gov/pubmed/?term=Ikawa%20F%5BAuthor%5D&cauthor=true&cauthor_uid=8672308), [Takeshita S](https://www.ncbi.nlm.nih.gov/pubmed/?term=Takeshita%20S%5BAuthor%5D&cauthor=true&cauthor_uid=8672308), [Hamasaki O](https://www.ncbi.nlm.nih.gov/pubmed/?term=Hamasaki%20O%5BAuthor%5D&cauthor=true&cauthor_uid=8672308), [Tohru U](https://www.ncbi.nlm.nih.gov/pubmed/?term=Tohru%20U%5BAuthor%5D&cauthor=true&cauthor_uid=8672308). A case of Rathke's cleft cyst in association with anterior communicating artery aneurysm presenting a rare visual field defect. [*No To Shinkei.*](https://www.ncbi.nlm.nih.gov/pubmed/8672308)*1*996;**48**(5):482-5.
26. [Li ZS](https://www.ncbi.nlm.nih.gov/pubmed/?term=Li%20ZS%5BAuthor%5D&cauthor=true&cauthor_uid=20497342), [Wei MQ](https://www.ncbi.nlm.nih.gov/pubmed/?term=Wei%20MQ%5BAuthor%5D&cauthor=true&cauthor_uid=20497342), [Fu X](https://www.ncbi.nlm.nih.gov/pubmed/?term=Fu%20X%5BAuthor%5D&cauthor=true&cauthor_uid=20497342), [Cheng H](https://www.ncbi.nlm.nih.gov/pubmed/?term=Cheng%20H%5BAuthor%5D&cauthor=true&cauthor_uid=20497342), [Li Q](https://www.ncbi.nlm.nih.gov/pubmed/?term=Li%20Q%5BAuthor%5D&cauthor=true&cauthor_uid=20497342). Chordoma coexisting with Rathke's cleft cyst: case report and literature review*.* [*Neuropathology.*](https://www.ncbi.nlm.nih.gov/pubmed/20497342) 2011;**31**(1):66-70.
27. [Wang K](https://www.ncbi.nlm.nih.gov/pubmed/?term=Wang%20K%5BAuthor%5D&cauthor=true&cauthor_uid=22824690), [Ma L](https://www.ncbi.nlm.nih.gov/pubmed/?term=Ma%20L%5BAuthor%5D&cauthor=true&cauthor_uid=22824690), [You C](https://www.ncbi.nlm.nih.gov/pubmed/?term=You%20C%5BAuthor%5D&cauthor=true&cauthor_uid=22824690). Pituitary adenoma and concomitant Rathke's cleft cyst: a case report and review of the literature. [*Neurol India.*](https://www.ncbi.nlm.nih.gov/pubmed/22824690) 2012;**60**(3):309-10.
28. [Radhakrishnan N](https://www.ncbi.nlm.nih.gov/pubmed/?term=Radhakrishnan%20N%5BAuthor%5D&cauthor=true&cauthor_uid=21934255), [Menon G](https://www.ncbi.nlm.nih.gov/pubmed/?term=Menon%20G%5BAuthor%5D&cauthor=true&cauthor_uid=21934255), [Hingwala DR](https://www.ncbi.nlm.nih.gov/pubmed/?term=Hingwala%20DR%5BAuthor%5D&cauthor=true&cauthor_uid=21934255), [Radhakrishnan VV](https://www.ncbi.nlm.nih.gov/pubmed/?term=Radhakrishnan%20VV%5BAuthor%5D&cauthor=true&cauthor_uid=21934255). Non-functioning pituitary adenoma and concomitant Rathke's cleft cyst. [*Indian J Pathol Microbiol.*](https://www.ncbi.nlm.nih.gov/pubmed/21934255) 2011; **54**(3): 649-51.
29. [Nishioka H](https://www.ncbi.nlm.nih.gov/pubmed/?term=Nishioka%20H%5BAuthor%5D&cauthor=true&cauthor_uid=10602857), [Ito H](https://www.ncbi.nlm.nih.gov/pubmed/?term=Ito%20H%5BAuthor%5D&cauthor=true&cauthor_uid=10602857), [Miki T](https://www.ncbi.nlm.nih.gov/pubmed/?term=Miki%20T%5BAuthor%5D&cauthor=true&cauthor_uid=10602857), [Hashimoto T](https://www.ncbi.nlm.nih.gov/pubmed/?term=Hashimoto%20T%5BAuthor%5D&cauthor=true&cauthor_uid=10602857), [Nojima H](https://www.ncbi.nlm.nih.gov/pubmed/?term=Nojima%20H%5BAuthor%5D&cauthor=true&cauthor_uid=10602857), [Matsumura H](https://www.ncbi.nlm.nih.gov/pubmed/?term=Matsumura%20H%5BAuthor%5D&cauthor=true&cauthor_uid=10602857). Rathke's cleft cyst with pituitary apoplexy: case report. [*Neuroradiology.*](https://www.ncbi.nlm.nih.gov/pubmed/10602857) 1999;**41**(11):832-4.
30. [Sonnet E](https://www.ncbi.nlm.nih.gov/pubmed/?term=Sonnet%20E%5BAuthor%5D&cauthor=true&cauthor_uid=16699303), [Roudaut N](https://www.ncbi.nlm.nih.gov/pubmed/?term=Roudaut%20N%5BAuthor%5D&cauthor=true&cauthor_uid=16699303), [Mériot P](https://www.ncbi.nlm.nih.gov/pubmed/?term=M%C3%A9riot%20P%5BAuthor%5D&cauthor=true&cauthor_uid=16699303), [Besson G](https://www.ncbi.nlm.nih.gov/pubmed/?term=Besson%20G%5BAuthor%5D&cauthor=true&cauthor_uid=16699303), [Kerlan V](https://www.ncbi.nlm.nih.gov/pubmed/?term=Kerlan%20V%5BAuthor%5D&cauthor=true&cauthor_uid=16699303). Hypophysitis associated with a ruptured Rathke's cleft cyst in a woman, during pregnancy. [*J Endocrinol Invest.*](https://www.ncbi.nlm.nih.gov/pubmed/?term=Sonnet+E%2C2006) 2006;**29**(4):353-7.
31. [Daikokuya H](https://www.ncbi.nlm.nih.gov/pubmed/?term=Daikokuya%20H%5BAuthor%5D&cauthor=true&cauthor_uid=10952189), [Inoue Y](https://www.ncbi.nlm.nih.gov/pubmed/?term=Inoue%20Y%5BAuthor%5D&cauthor=true&cauthor_uid=10952189), [Nemoto Y](https://www.ncbi.nlm.nih.gov/pubmed/?term=Nemoto%20Y%5BAuthor%5D&cauthor=true&cauthor_uid=10952189), [Tashiro T](https://www.ncbi.nlm.nih.gov/pubmed/?term=Tashiro%20T%5BAuthor%5D&cauthor=true&cauthor_uid=10952189), [Shakudo M](https://www.ncbi.nlm.nih.gov/pubmed/?term=Shakudo%20M%5BAuthor%5D&cauthor=true&cauthor_uid=10952189), [Ohata K](https://www.ncbi.nlm.nih.gov/pubmed/?term=Ohata%20K%5BAuthor%5D&cauthor=true&cauthor_uid=10952189). Rathke's cleft cyst associated with hypophysitis: MRI. [*Neuroradiology.*](https://www.ncbi.nlm.nih.gov/pubmed/10952189) 2000;**42**(7):532-4.
32. [Uchiyama T](https://www.ncbi.nlm.nih.gov/pubmed/?term=Uchiyama%20T%5BAuthor%5D&cauthor=true&cauthor_uid=21701113), [Sakai K](https://www.ncbi.nlm.nih.gov/pubmed/?term=Sakai%20K%5BAuthor%5D&cauthor=true&cauthor_uid=21701113), [Asanuma M](https://www.ncbi.nlm.nih.gov/pubmed/?term=Asanuma%20M%5BAuthor%5D&cauthor=true&cauthor_uid=21701113), [Aoyama T](https://www.ncbi.nlm.nih.gov/pubmed/?term=Aoyama%20T%5BAuthor%5D&cauthor=true&cauthor_uid=21701113), [Hongo K](https://www.ncbi.nlm.nih.gov/pubmed/?term=Hongo%20K%5BAuthor%5D&cauthor=true&cauthor_uid=21701113). Pituitary abscess manifesting as meningitis and photophobia associated with Rathke's cleft cyst in a child. Case report. [*Neurol Med Chir (Tokyo).*](https://www.ncbi.nlm.nih.gov/pubmed/21701113) 2011;**51**(6):455-9.
33. [Alomari AK](https://www.ncbi.nlm.nih.gov/pubmed/?term=Alomari%20AK%5BAuthor%5D&cauthor=true&cauthor_uid=25555112), [Kelley BJ](https://www.ncbi.nlm.nih.gov/pubmed/?term=Kelley%20BJ%5BAuthor%5D&cauthor=true&cauthor_uid=25555112), [Damisah E](https://www.ncbi.nlm.nih.gov/pubmed/?term=Damisah%20E%5BAuthor%5D&cauthor=true&cauthor_uid=25555112), [Marks A](https://www.ncbi.nlm.nih.gov/pubmed/?term=Marks%20A%5BAuthor%5D&cauthor=true&cauthor_uid=25555112), [Hui P](https://www.ncbi.nlm.nih.gov/pubmed/?term=Hui%20P%5BAuthor%5D&cauthor=true&cauthor_uid=25555112), [DiLuna M](https://www.ncbi.nlm.nih.gov/pubmed/?term=DiLuna%20M%5BAuthor%5D&cauthor=true&cauthor_uid=25555112), [Vortmeyer A](https://www.ncbi.nlm.nih.gov/pubmed/?term=Vortmeyer%20A%5BAuthor%5D&cauthor=true&cauthor_uid=25555112). Craniopharyngioma arising in a Rathke's cleft cyst: case report. [*J Neurosurg Pediatr.*](https://www.ncbi.nlm.nih.gov/pubmed/25555112) 2015;**15**(3):250-4.
34. [Hama S](https://www.ncbi.nlm.nih.gov/pubmed/?term=Hama%20S%5BAuthor%5D&cauthor=true&cauthor_uid=10426586), [Arita K](https://www.ncbi.nlm.nih.gov/pubmed/?term=Arita%20K%5BAuthor%5D&cauthor=true&cauthor_uid=10426586), [Tominaga A](https://www.ncbi.nlm.nih.gov/pubmed/?term=Tominaga%20A%5BAuthor%5D&cauthor=true&cauthor_uid=10426586), [Yoshikawa M](https://www.ncbi.nlm.nih.gov/pubmed/?term=Yoshikawa%20M%5BAuthor%5D&cauthor=true&cauthor_uid=10426586), [Eguchi K](https://www.ncbi.nlm.nih.gov/pubmed/?term=Eguchi%20K%5BAuthor%5D&cauthor=true&cauthor_uid=10426586), [Sumida M](https://www.ncbi.nlm.nih.gov/pubmed/?term=Sumida%20M%5BAuthor%5D&cauthor=true&cauthor_uid=10426586), [Inai K](https://www.ncbi.nlm.nih.gov/pubmed/?term=Inai%20K%5BAuthor%5D&cauthor=true&cauthor_uid=10426586), [Nishisaka T](https://www.ncbi.nlm.nih.gov/pubmed/?term=Nishisaka%20T%5BAuthor%5D&cauthor=true&cauthor_uid=10426586), [Kurisu K](https://www.ncbi.nlm.nih.gov/pubmed/?term=Kurisu%20K%5BAuthor%5D&cauthor=true&cauthor_uid=10426586). Symptomatic Rathke's cleft cyst coexisting with central diabetes insipidus and hypophysitis: case report. [*Endocr J*.](https://www.ncbi.nlm.nih.gov/pubmed/10426586) 1999;**46**(1):187-92.
35. [Takahashi D](https://www.ncbi.nlm.nih.gov/pubmed/?term=Takahashi%20D%5BAuthor%5D&cauthor=true&cauthor_uid=23729539), [Kobayashi H](https://www.ncbi.nlm.nih.gov/pubmed/?term=Kobayashi%20H%5BAuthor%5D&cauthor=true&cauthor_uid=23729539), [Kubota K](https://www.ncbi.nlm.nih.gov/pubmed/?term=Kubota%20K%5BAuthor%5D&cauthor=true&cauthor_uid=23729539), [Suzuki Y](https://www.ncbi.nlm.nih.gov/pubmed/?term=Suzuki%20Y%5BAuthor%5D&cauthor=true&cauthor_uid=23729539), [Nakamura A](https://www.ncbi.nlm.nih.gov/pubmed/?term=Nakamura%20A%5BAuthor%5D&cauthor=true&cauthor_uid=23729539), [Ishizu K](https://www.ncbi.nlm.nih.gov/pubmed/?term=Ishizu%20K%5BAuthor%5D&cauthor=true&cauthor_uid=23729539), [Nakanishi M](https://www.ncbi.nlm.nih.gov/pubmed/?term=Nakanishi%20M%5BAuthor%5D&cauthor=true&cauthor_uid=23729539), [Nagashima T](https://www.ncbi.nlm.nih.gov/pubmed/?term=Nagashima%20T%5BAuthor%5D&cauthor=true&cauthor_uid=23729539), [Tajima T](https://www.ncbi.nlm.nih.gov/pubmed/?term=Tajima%20T%5BAuthor%5D&cauthor=true&cauthor_uid=23729539). A rare association between Rathke's cyst and hypophysitis in a patient with delayed sex development and growth failure. [*J Pediatr Endocrinol Metab.*](https://www.ncbi.nlm.nih.gov/pubmed/23729539) 2013;**26**(9-10):949-53.
36. [Sakamoto S](https://www.ncbi.nlm.nih.gov/pubmed/?term=Sakamoto%20S%5BAuthor%5D&cauthor=true&cauthor_uid=11857945), [Ikawa F](https://www.ncbi.nlm.nih.gov/pubmed/?term=Ikawa%20F%5BAuthor%5D&cauthor=true&cauthor_uid=11857945), [Kawamoto H](https://www.ncbi.nlm.nih.gov/pubmed/?term=Kawamoto%20H%5BAuthor%5D&cauthor=true&cauthor_uid=11857945), [Ohbayashi N](https://www.ncbi.nlm.nih.gov/pubmed/?term=Ohbayashi%20N%5BAuthor%5D&cauthor=true&cauthor_uid=11857945), [Sakoda E](https://www.ncbi.nlm.nih.gov/pubmed/?term=Sakoda%20E%5BAuthor%5D&cauthor=true&cauthor_uid=11857945), [Hidaka T](https://www.ncbi.nlm.nih.gov/pubmed/?term=Hidaka%20T%5BAuthor%5D&cauthor=true&cauthor_uid=11857945), [Inagawa T](https://www.ncbi.nlm.nih.gov/pubmed/?term=Inagawa%20T%5BAuthor%5D&cauthor=true&cauthor_uid=11857945). A case of Rathke's cleft cyst in association with a ruptured aneurysm of the anterior cerebral artery mimicking pituitary apoplexy. [*No Shinkei Geka*.](https://www.ncbi.nlm.nih.gov/pubmed/11857945) 2002;**30**(2):199-203.
37. [Albini CH](https://www.ncbi.nlm.nih.gov/pubmed/?term=Albini%20CH%5BAuthor%5D&cauthor=true&cauthor_uid=3344071), [MacGillivray MH](https://www.ncbi.nlm.nih.gov/pubmed/?term=MacGillivray%20MH%5BAuthor%5D&cauthor=true&cauthor_uid=3344071), [Fisher JE](https://www.ncbi.nlm.nih.gov/pubmed/?term=Fisher%20JE%5BAuthor%5D&cauthor=true&cauthor_uid=3344071), [Voorhess ML](https://www.ncbi.nlm.nih.gov/pubmed/?term=Voorhess%20ML%5BAuthor%5D&cauthor=true&cauthor_uid=3344071), [Klein DM](https://www.ncbi.nlm.nih.gov/pubmed/?term=Klein%20DM%5BAuthor%5D&cauthor=true&cauthor_uid=3344071). Triad of hypopituitarism, granulomatous hypophysitis, and ruptured Rathke's cleft cyst. [*Neurosurgery.*](https://www.ncbi.nlm.nih.gov/pubmed/3344071) 1988;**22**(1 Pt 1):133-6.
38. [Yang C](https://www.ncbi.nlm.nih.gov/pubmed/?term=Yang%20C%5BAuthor%5D&cauthor=true&cauthor_uid=29572168), [Wu H](https://www.ncbi.nlm.nih.gov/pubmed/?term=Wu%20H%5BAuthor%5D&cauthor=true&cauthor_uid=29572168), [Bao X](https://www.ncbi.nlm.nih.gov/pubmed/?term=Bao%20X%5BAuthor%5D&cauthor=true&cauthor_uid=29572168), [Wang R](https://www.ncbi.nlm.nih.gov/pubmed/?term=Wang%20R%5BAuthor%5D&cauthor=true&cauthor_uid=29572168). Lymphocytic Hypophysitis Secondary to Ruptured Rathke Cleft Cyst: Case Report and Literature Review. [*World Neurosurg.*](https://www.ncbi.nlm.nih.gov/pubmed/29572168) 2018 ;**114**:172-177.
39. [Draghi R](https://www.ncbi.nlm.nih.gov/pubmed/?term=Draghi%20R%5BAuthor%5D&cauthor=true&cauthor_uid=29552263), [Mantovani G](https://www.ncbi.nlm.nih.gov/pubmed/?term=Mantovani%20G%5BAuthor%5D&cauthor=true&cauthor_uid=29552263), [Runza L](https://www.ncbi.nlm.nih.gov/pubmed/?term=Runza%20L%5BAuthor%5D&cauthor=true&cauthor_uid=29552263), [Carrabba G](https://www.ncbi.nlm.nih.gov/pubmed/?term=Carrabba%20G%5BAuthor%5D&cauthor=true&cauthor_uid=29552263), [Fusco N](https://www.ncbi.nlm.nih.gov/pubmed/?term=Fusco%20N%5BAuthor%5D&cauthor=true&cauthor_uid=29552263), [Rampini P](https://www.ncbi.nlm.nih.gov/pubmed/?term=Rampini%20P%5BAuthor%5D&cauthor=true&cauthor_uid=29552263), [Costa A](https://www.ncbi.nlm.nih.gov/pubmed/?term=Costa%20A%5BAuthor%5D&cauthor=true&cauthor_uid=29552263), [Locatelli M](https://www.ncbi.nlm.nih.gov/pubmed/?term=Locatelli%20M%5BAuthor%5D&cauthor=true&cauthor_uid=29552263). Rathke's cleft cyst associated with pituitary granulomatosis with polyangiitis: An unusual combination of hypothalamus-pituitary region pathologies. [*Radiol Case Rep.*](https://www.ncbi.nlm.nih.gov/pubmed/29552263) 2017; 31;**13**(1):233-236.
40. [Gupta V](https://www.ncbi.nlm.nih.gov/pubmed/?term=Gupta%20V%5BAuthor%5D&cauthor=true&cauthor_uid=21731878), [Grossman A](https://www.ncbi.nlm.nih.gov/pubmed/?term=Grossman%20A%5BAuthor%5D&cauthor=true&cauthor_uid=21731878), [Kapadia A](https://www.ncbi.nlm.nih.gov/pubmed/?term=Kapadia%20A%5BAuthor%5D&cauthor=true&cauthor_uid=21731878), [Thorat K](https://www.ncbi.nlm.nih.gov/pubmed/?term=Thorat%20K%5BAuthor%5D&cauthor=true&cauthor_uid=21731878). Acromegaly associated with a symptomatic Rathke's cyst. [*Indian J Endocrinol Metab.*](https://www.ncbi.nlm.nih.gov/pubmed/21731878) 2011;**15**(2):140-2.
41. [Nishimura F](https://www.ncbi.nlm.nih.gov/pubmed/?term=Nishimura%20F%5BAuthor%5D&cauthor=true&cauthor_uid=30610983), [Park YS](https://www.ncbi.nlm.nih.gov/pubmed/?term=Park%20YS%5BAuthor%5D&cauthor=true&cauthor_uid=30610983), [Motoyama Y](https://www.ncbi.nlm.nih.gov/pubmed/?term=Motoyama%20Y%5BAuthor%5D&cauthor=true&cauthor_uid=30610983), [Nakagawa I](https://www.ncbi.nlm.nih.gov/pubmed/?term=Nakagawa%20I%5BAuthor%5D&cauthor=true&cauthor_uid=30610983), [Yamada S](https://www.ncbi.nlm.nih.gov/pubmed/?term=Yamada%20S%5BAuthor%5D&cauthor=true&cauthor_uid=30610983), [Tamura K](https://www.ncbi.nlm.nih.gov/pubmed/?term=Tamura%20K%5BAuthor%5D&cauthor=true&cauthor_uid=30610983), [Matsuda R](https://www.ncbi.nlm.nih.gov/pubmed/?term=Matsuda%20R%5BAuthor%5D&cauthor=true&cauthor_uid=30610983), [Takeshima Y](https://www.ncbi.nlm.nih.gov/pubmed/?term=Takeshima%20Y%5BAuthor%5D&cauthor=true&cauthor_uid=30610983), [Takamura Y](https://www.ncbi.nlm.nih.gov/pubmed/?term=Takamura%20Y%5BAuthor%5D&cauthor=true&cauthor_uid=30610983), [Nakase H](https://www.ncbi.nlm.nih.gov/pubmed/?term=Nakase%20H%5BAuthor%5D&cauthor=true&cauthor_uid=30610983). Intractable Rathke's cleft cyst hidden behind co-existing giant pituitary adenoma - Case report. [*World Neurosurg.*](https://www.ncbi.nlm.nih.gov/pubmed/30610983) 2019;**2**. pii: S1878-8750(18):32928-0.
42. [Babu R](https://www.ncbi.nlm.nih.gov/pubmed/?term=Babu%20R%5BAuthor%5D&cauthor=true&cauthor_uid=24551002), [Back AG](https://www.ncbi.nlm.nih.gov/pubmed/?term=Back%20AG%5BAuthor%5D&cauthor=true&cauthor_uid=24551002), [Komisarow JM](https://www.ncbi.nlm.nih.gov/pubmed/?term=Komisarow%20JM%5BAuthor%5D&cauthor=true&cauthor_uid=24551002), [Owens TR](https://www.ncbi.nlm.nih.gov/pubmed/?term=Owens%20TR%5BAuthor%5D&cauthor=true&cauthor_uid=24551002), [Cummings TJ](https://www.ncbi.nlm.nih.gov/pubmed/?term=Cummings%20TJ%5BAuthor%5D&cauthor=true&cauthor_uid=24551002), [Britz GW](https://www.ncbi.nlm.nih.gov/pubmed/?term=Britz%20GW%5BAuthor%5D&cauthor=true&cauthor_uid=24551002).Symptomatic Rathke's cleft cyst with a co-existing pituitary tumor; Brief review of the literature. [*Asian J Neurosurg.*](https://www.ncbi.nlm.nih.gov/pubmed/24551002) 2013; **8** (4): 183-7.
43. [Tang C](https://www.ncbi.nlm.nih.gov/pubmed/?term=Tang%20C%5BAuthor%5D&cauthor=true&cauthor_uid=30080768), [Qiao L](https://www.ncbi.nlm.nih.gov/pubmed/?term=Qiao%20L%5BAuthor%5D&cauthor=true&cauthor_uid=30080768), [Zhong C](https://www.ncbi.nlm.nih.gov/pubmed/?term=Zhong%20C%5BAuthor%5D&cauthor=true&cauthor_uid=30080768), [Yang J](https://www.ncbi.nlm.nih.gov/pubmed/?term=Yang%20J%5BAuthor%5D&cauthor=true&cauthor_uid=30080768), [Zhu J](https://www.ncbi.nlm.nih.gov/pubmed/?term=Zhu%20J%5BAuthor%5D&cauthor=true&cauthor_uid=30080768), [Ma C](https://www.ncbi.nlm.nih.gov/pubmed/?term=Ma%20C%5BAuthor%5D&cauthor=true&cauthor_uid=30080768). The Coexistence of Growth Hormone-Producing Pituitary Adenoma and Rathke Cleft Cyst: How Can We Diagnosis Preoperation? [*J Craniofac Surg.*](https://www.ncbi.nlm.nih.gov/pubmed/30080768) 2018 ;**29**(7):1887-1889.
44. [Zhou P](https://www.ncbi.nlm.nih.gov/pubmed/?term=Zhou%20P%5BAuthor%5D&cauthor=true&cauthor_uid=23287345), [Cai B](https://www.ncbi.nlm.nih.gov/pubmed/?term=Cai%20B%5BAuthor%5D&cauthor=true&cauthor_uid=23287345), [Ma W](https://www.ncbi.nlm.nih.gov/pubmed/?term=Ma%20W%5BAuthor%5D&cauthor=true&cauthor_uid=23287345), [Jiang S](https://www.ncbi.nlm.nih.gov/pubmed/?term=Jiang%20S%5BAuthor%5D&cauthor=true&cauthor_uid=23287345). Combined pituitary adenoma and Rathke's cleft cysts: two multicystic cases and literature review. [*Neurol India.*](https://www.ncbi.nlm.nih.gov/pubmed/23287345) 2012;**60**(6):665-7.

1. [Satoshi Nakasu.](https://www.sciencedirect.com/science/article/pii/0090301989902164#!)[Yoko Nakasu.](https://www.sciencedirect.com/science/article/pii/0090301989902164#!) [Kazumitu Kyoshima.](https://www.sciencedirect.com/science/article/pii/0090301989902164#!) [Kazuyoshi Watanabe.](https://www.sciencedirect.com/science/article/pii/0090301989902164#!) [Jyoji Handa.](https://www.sciencedirect.com/science/article/pii/0090301989902164#!) [Hidetoshi Okabe](https://www.sciencedirect.com/science/article/pii/0090301989902164#!). Pituitary adenoma with multiple ciliated cysts: Transitional cell tumor? [*Surgical Neurology*](https://www.sciencedirect.com/science/journal/00903019)*.* 1989; [**31**(1](https://www.sciencedirect.com/science/journal/00903019/31/1)):41-48.
2. [Swanson SE](https://www.ncbi.nlm.nih.gov/pubmed/?term=Swanson%20SE%5BAuthor%5D&cauthor=true&cauthor_uid=4058703), [Chandler WF](https://www.ncbi.nlm.nih.gov/pubmed/?term=Chandler%20WF%5BAuthor%5D&cauthor=true&cauthor_uid=4058703), [Latack J](https://www.ncbi.nlm.nih.gov/pubmed/?term=Latack%20J%5BAuthor%5D&cauthor=true&cauthor_uid=4058703), [Zis K](https://www.ncbi.nlm.nih.gov/pubmed/?term=Zis%20K%5BAuthor%5D&cauthor=true&cauthor_uid=4058703). Symptomatic Rathke's cleft cyst with pituitary adenoma: case report. [*Neurosurgery.*](https://www.ncbi.nlm.nih.gov/pubmed/4058703) 1985;**17**(4):657-9.
3. [Kepes JJ](https://www.ncbi.nlm.nih.gov/pubmed/?term=Kepes%20JJ%5BAuthor%5D&cauthor=true&cauthor_uid=626939). Transitional cell tumor of the pituitary gland developing from a Rathke's cleft cyst. [*Cancer.*](https://www.ncbi.nlm.nih.gov/pubmed/626939)*1*978;**41** (1):337-43.
4. [Brassier G](https://www.ncbi.nlm.nih.gov/pubmed/?term=Brassier%20G%5BAuthor%5D&cauthor=true&cauthor_uid=10655672), [Morandi X](https://www.ncbi.nlm.nih.gov/pubmed/?term=Morandi%20X%5BAuthor%5D&cauthor=true&cauthor_uid=10655672), [Tayiar E](https://www.ncbi.nlm.nih.gov/pubmed/?term=Tayiar%20E%5BAuthor%5D&cauthor=true&cauthor_uid=10655672), [Riffaud L](https://www.ncbi.nlm.nih.gov/pubmed/?term=Riffaud%20L%5BAuthor%5D&cauthor=true&cauthor_uid=10655672), [Chabert E](https://www.ncbi.nlm.nih.gov/pubmed/?term=Chabert%20E%5BAuthor%5D&cauthor=true&cauthor_uid=10655672), [Heresbach N](https://www.ncbi.nlm.nih.gov/pubmed/?term=Heresbach%20N%5BAuthor%5D&cauthor=true&cauthor_uid=10655672), [Poirier JY](https://www.ncbi.nlm.nih.gov/pubmed/?term=Poirier%20JY%5BAuthor%5D&cauthor=true&cauthor_uid=10655672), [Carsin-Nicol B](https://www.ncbi.nlm.nih.gov/pubmed/?term=Carsin-Nicol%20B%5BAuthor%5D&cauthor=true&cauthor_uid=10655672). Rathke's cleft cysts: surgical-MRI correlation in 16 symptomatic cases. [*J Neuroradiol*.](https://www.ncbi.nlm.nih.gov/pubmed/10655672) 1999;**26** (3):162-71.
5. [Amano T](https://www.ncbi.nlm.nih.gov/pubmed/?term=Amano%20T%5BAuthor%5D&cauthor=true&cauthor_uid=14978924), [Kajiwara K](https://www.ncbi.nlm.nih.gov/pubmed/?term=Kajiwara%20K%5BAuthor%5D&cauthor=true&cauthor_uid=14978924), [Harada K](https://www.ncbi.nlm.nih.gov/pubmed/?term=Harada%20K%5BAuthor%5D&cauthor=true&cauthor_uid=14978924), [Yoshikawa K](https://www.ncbi.nlm.nih.gov/pubmed/?term=Yoshikawa%20K%5BAuthor%5D&cauthor=true&cauthor_uid=14978924), [Akimura T](https://www.ncbi.nlm.nih.gov/pubmed/?term=Akimura%20T%5BAuthor%5D&cauthor=true&cauthor_uid=14978924), [Kato S](https://www.ncbi.nlm.nih.gov/pubmed/?term=Kato%20S%5BAuthor%5D&cauthor=true&cauthor_uid=14978924), [Fujii M](https://www.ncbi.nlm.nih.gov/pubmed/?term=Fujii%20M%5BAuthor%5D&cauthor=true&cauthor_uid=14978924), [Fujisawa H](https://www.ncbi.nlm.nih.gov/pubmed/?term=Fujisawa%20H%5BAuthor%5D&cauthor=true&cauthor_uid=14978924), [Suzuki M](https://www.ncbi.nlm.nih.gov/pubmed/?term=Suzuki%20M%5BAuthor%5D&cauthor=true&cauthor_uid=14978924). A case of Rathke's cleft cyst in association with bilateral unruptured aneurysms of internal carotid artery. [*No Shinkei Geka.*](https://www.ncbi.nlm.nih.gov/pubmed/14978924) 2004 ;**32**(1):49-54.
6. [Ikeda H](https://www.ncbi.nlm.nih.gov/pubmed/?term=Ikeda%20H%5BAuthor%5D&cauthor=true&cauthor_uid=3320806), [Niizuma H](https://www.ncbi.nlm.nih.gov/pubmed/?term=Niizuma%20H%5BAuthor%5D&cauthor=true&cauthor_uid=3320806), [Fujiwara S](https://www.ncbi.nlm.nih.gov/pubmed/?term=Fujiwara%20S%5BAuthor%5D&cauthor=true&cauthor_uid=3320806), [Suzuki J](https://www.ncbi.nlm.nih.gov/pubmed/?term=Suzuki%20J%5BAuthor%5D&cauthor=true&cauthor_uid=3320806), [Sasano N](https://www.ncbi.nlm.nih.gov/pubmed/?term=Sasano%20N%5BAuthor%5D&cauthor=true&cauthor_uid=3320806). A case of prolactinoma in close association with Rathke's cleft cyst. [*No Shinkei Geka.*](https://www.ncbi.nlm.nih.gov/pubmed/3320806) 1987;**15(**9):999-1003.
7. [Matsumori K](https://www.ncbi.nlm.nih.gov/pubmed/?term=Matsumori%20K%5BAuthor%5D&cauthor=true&cauthor_uid=6483092), [Okuda T](https://www.ncbi.nlm.nih.gov/pubmed/?term=Okuda%20T%5BAuthor%5D&cauthor=true&cauthor_uid=6483092), [Nakayama K](https://www.ncbi.nlm.nih.gov/pubmed/?term=Nakayama%20K%5BAuthor%5D&cauthor=true&cauthor_uid=6483092), [Miyasaka Y](https://www.ncbi.nlm.nih.gov/pubmed/?term=Miyasaka%20Y%5BAuthor%5D&cauthor=true&cauthor_uid=6483092), [Beppu T](https://www.ncbi.nlm.nih.gov/pubmed/?term=Beppu%20T%5BAuthor%5D&cauthor=true&cauthor_uid=6483092), [Kubo O](https://www.ncbi.nlm.nih.gov/pubmed/?term=Kubo%20O%5BAuthor%5D&cauthor=true&cauthor_uid=6483092). Case of calcified prolactinoma combined with Rathke's cleft cysts. [*No Shinkei Geka.*](https://www.ncbi.nlm.nih.gov/pubmed/6483092) 1984 ;**12**(7):833-8.
8. [Trokoudes KM](https://www.ncbi.nlm.nih.gov/pubmed/?term=Trokoudes%20KM%5BAuthor%5D&cauthor=true&cauthor_uid=660898), [Walfish PG](https://www.ncbi.nlm.nih.gov/pubmed/?term=Walfish%20PG%5BAuthor%5D&cauthor=true&cauthor_uid=660898), [Holgate RC](https://www.ncbi.nlm.nih.gov/pubmed/?term=Holgate%20RC%5BAuthor%5D&cauthor=true&cauthor_uid=660898), [Pritzker KP](https://www.ncbi.nlm.nih.gov/pubmed/?term=Pritzker%20KP%5BAuthor%5D&cauthor=true&cauthor_uid=660898), [Schwartz ML](https://www.ncbi.nlm.nih.gov/pubmed/?term=Schwartz%20ML%5BAuthor%5D&cauthor=true&cauthor_uid=660898), [Kovacs K](https://www.ncbi.nlm.nih.gov/pubmed/?term=Kovacs%20K%5BAuthor%5D&cauthor=true&cauthor_uid=660898). Sellar enlargement with hyperprolactinemia and a Rathke's pouch cyst. [*JAMA.*](https://www.ncbi.nlm.nih.gov/pubmed/660898) 1978; 4;**240**(5):471-3.
9. [Karavitaki N](https://www.ncbi.nlm.nih.gov/pubmed/?term=Karavitaki%20N%5BAuthor%5D&cauthor=true&cauthor_uid=17917812), [Scheithauer BW](https://www.ncbi.nlm.nih.gov/pubmed/?term=Scheithauer%20BW%5BAuthor%5D&cauthor=true&cauthor_uid=17917812), [Watt J](https://www.ncbi.nlm.nih.gov/pubmed/?term=Watt%20J%5BAuthor%5D&cauthor=true&cauthor_uid=17917812), [Ansorge O](https://www.ncbi.nlm.nih.gov/pubmed/?term=Ansorge%20O%5BAuthor%5D&cauthor=true&cauthor_uid=17917812), [Moschopoulos M](https://www.ncbi.nlm.nih.gov/pubmed/?term=Moschopoulos%20M%5BAuthor%5D&cauthor=true&cauthor_uid=17917812), [Llaguno AV](https://www.ncbi.nlm.nih.gov/pubmed/?term=Llaguno%20AV%5BAuthor%5D&cauthor=true&cauthor_uid=17917812), [Wass JA](https://www.ncbi.nlm.nih.gov/pubmed/?term=Wass%20JA%5BAuthor%5D&cauthor=true&cauthor_uid=17917812). Collision lesions of the sella: co-existence of craniopharyngioma with gonadotroph adenoma and of Rathke's cleft cyst with corticotroph adenoma. [*Pituitary.*](https://www.ncbi.nlm.nih.gov/pubmed/17917812) 2008;**11**(3):317-23.
10. [Tamura R](https://www.ncbi.nlm.nih.gov/pubmed/?term=Tamura%20R%5BAuthor%5D&cauthor=true&cauthor_uid=25883817), [Takahashi S](https://www.ncbi.nlm.nih.gov/pubmed/?term=Takahashi%20S%5BAuthor%5D&cauthor=true&cauthor_uid=25883817), [Emoto K](https://www.ncbi.nlm.nih.gov/pubmed/?term=Emoto%20K%5BAuthor%5D&cauthor=true&cauthor_uid=25883817), [Nagashima H](https://www.ncbi.nlm.nih.gov/pubmed/?term=Nagashima%20H%5BAuthor%5D&cauthor=true&cauthor_uid=25883817), [Toda M](https://www.ncbi.nlm.nih.gov/pubmed/?term=Toda%20M%5BAuthor%5D&cauthor=true&cauthor_uid=25883817). [Yoshida K](https://www.ncbi.nlm.nih.gov/pubmed/?term=Yoshida%20K%5BAuthor%5D&cauthor=true&cauthor_uid=25883817). GH-Producing Pituitary Adenoma and Concomitant Rathke's Cleft Cyst: A Case Report and Short Review. [*Case Rep Neurol Med.*](https://www.ncbi.nlm.nih.gov/pubmed/25883817) 2015;948025.
11. [Shuangshoti S](https://www.ncbi.nlm.nih.gov/pubmed/?term=Shuangshoti%20S%5BAuthor%5D&cauthor=true&cauthor_uid=5476241), [Netsky MG](https://www.ncbi.nlm.nih.gov/pubmed/?term=Netsky%20MG%5BAuthor%5D&cauthor=true&cauthor_uid=5476241), [Nashold BS Jr](https://www.ncbi.nlm.nih.gov/pubmed/?term=Nashold%20BS%20Jr%5BAuthor%5D&cauthor=true&cauthor_uid=5476241). Epithelial cysts related to sella turcica. Proposed origin from neuroepithelium. [*Arch Pathol.*](https://www.ncbi.nlm.nih.gov/pubmed/5476241) 1970;**90**(5):444-50.
12. [Duffy WC](https://www.ncbi.nlm.nih.gov/pubmed/?term=Duffy%20WC%5BAuthor%5D&cauthor=true&cauthor_uid=17864361). Hypophyseal duct tumors: A Report of Three case and A fourth case of cyst of Rathke’s pouch. [*Ann Surg*.](https://www.ncbi.nlm.nih.gov/pubmed/17864361) 1920;**72** (6):725-57.
13. [Latifaci I](https://www.ncbi.nlm.nih.gov/pubmed/?term=Latifaci%20I%5BAuthor%5D&cauthor=true&cauthor_uid=28701004), [Iplikcioglu C](https://www.ncbi.nlm.nih.gov/pubmed/?term=Iplikcioglu%20C%5BAuthor%5D&cauthor=true&cauthor_uid=28701004), [Tokmak M](https://www.ncbi.nlm.nih.gov/pubmed/?term=Tokmak%20M%5BAuthor%5D&cauthor=true&cauthor_uid=28701004), [Ozek E](https://www.ncbi.nlm.nih.gov/pubmed/?term=Ozek%20E%5BAuthor%5D&cauthor=true&cauthor_uid=28701004). En Plaque Pituitary Adenoma within a Rathke's Cleft Cyst: Report of Three Cases. [*J Neurol Surg A Cent Eur Neurosurg.*](https://www.ncbi.nlm.nih.gov/pubmed/28701004) 2018 ;**79** (1):86-89.
14. [Chaudhry NS](https://www.ncbi.nlm.nih.gov/pubmed/?term=Chaudhry%20NS%5BAuthor%5D&cauthor=true&cauthor_uid=27600168), [Raber MR](https://www.ncbi.nlm.nih.gov/pubmed/?term=Raber%20MR%5BAuthor%5D&cauthor=true&cauthor_uid=27600168), [Cote DJ](https://www.ncbi.nlm.nih.gov/pubmed/?term=Cote%20DJ%5BAuthor%5D&cauthor=true&cauthor_uid=27600168), [Laws ER Jr](https://www.ncbi.nlm.nih.gov/pubmed/?term=Laws%20ER%20Jr%5BAuthor%5D&cauthor=true&cauthor_uid=27600168). Spontaneous pituitary adenoma occurring after resection of a Rathke's cleft cyst. [J Clin Neurosci.](https://www.ncbi.nlm.nih.gov/pubmed/27600168) 2016;**33**:247-251.
15. [Wu W](https://www.ncbi.nlm.nih.gov/pubmed/?term=Wu%20W%5BAuthor%5D&cauthor=true&cauthor_uid=29103386), [Jia G](https://www.ncbi.nlm.nih.gov/pubmed/?term=Jia%20G%5BAuthor%5D&cauthor=true&cauthor_uid=29103386), [Jia W](https://www.ncbi.nlm.nih.gov/pubmed/?term=Jia%20W%5BAuthor%5D&cauthor=true&cauthor_uid=29103386), [Li G](https://www.ncbi.nlm.nih.gov/pubmed/?term=Li%20G%5BAuthor%5D&cauthor=true&cauthor_uid=29103386), [Zhang J](https://www.ncbi.nlm.nih.gov/pubmed/?term=Zhang%20J%5BAuthor%5D&cauthor=true&cauthor_uid=29103386), [Zhang L](https://www.ncbi.nlm.nih.gov/pubmed/?term=Zhang%20L%5BAuthor%5D&cauthor=true&cauthor_uid=29103386). Pituitary Adenoma Associated With Rathke's Cleft Cyst: Report of 15 Cases. [*Can J Neurol Sci.*](https://www.ncbi.nlm.nih.gov/pubmed/29103386) 2018;**45**(1):68-75.
16. [Manjila S](https://www.ncbi.nlm.nih.gov/pubmed/?term=Manjila%20S%5BAuthor%5D&cauthor=true&cauthor_uid=30010935), [Asmar NE](https://www.ncbi.nlm.nih.gov/pubmed/?term=Asmar%20NE%5BAuthor%5D&cauthor=true&cauthor_uid=30010935), [Vidalis BM](https://www.ncbi.nlm.nih.gov/pubmed/?term=Vidalis%20BM%5BAuthor%5D&cauthor=true&cauthor_uid=30010935), [Alonso F](https://www.ncbi.nlm.nih.gov/pubmed/?term=Alonso%20F%5BAuthor%5D&cauthor=true&cauthor_uid=30010935), [Singh G](https://www.ncbi.nlm.nih.gov/pubmed/?term=Singh%20G%5BAuthor%5D&cauthor=true&cauthor_uid=30010935), [Vadamalai K](https://www.ncbi.nlm.nih.gov/pubmed/?term=Vadamalai%20K%5BAuthor%5D&cauthor=true&cauthor_uid=30010935), [Cohen ML](https://www.ncbi.nlm.nih.gov/pubmed/?term=Cohen%20ML%5BAuthor%5D&cauthor=true&cauthor_uid=30010935), [Bambakidis NC](https://www.ncbi.nlm.nih.gov/pubmed/?term=Bambakidis%20NC%5BAuthor%5D&cauthor=true&cauthor_uid=30010935), [Arafah BM](https://www.ncbi.nlm.nih.gov/pubmed/?term=Arafah%20BM%5BAuthor%5D&cauthor=true&cauthor_uid=30010935), [Selman WR](https://www.ncbi.nlm.nih.gov/pubmed/?term=Selman%20WR%5BAuthor%5D&cauthor=true&cauthor_uid=30010935). Intratumoral Rathke's Cleft Cyst Remnants Within Craniopharyngioma, Pituitary Adenoma, Suprasellar Dermoid, and Epidermoid Cysts: A Ubiquitous Signature of Ectodermal Lineage or a Transitional Entity?  [*Neurosurgery*.](https://www.ncbi.nlm.nih.gov/pubmed/30010935) 2019; 85(2):180-188.
17. [Gessler F](https://www.ncbi.nlm.nih.gov/pubmed/?term=Gessler%20F%5BAuthor%5D&cauthor=true&cauthor_uid=23984210), [Coon VC](https://www.ncbi.nlm.nih.gov/pubmed/?term=Coon%20VC%5BAuthor%5D&cauthor=true&cauthor_uid=23984210), [Chin SS](https://www.ncbi.nlm.nih.gov/pubmed/?term=Chin%20SS%5BAuthor%5D&cauthor=true&cauthor_uid=23984210), [Couldwell WT](https://www.ncbi.nlm.nih.gov/pubmed/?term=Couldwell%20WT%5BAuthor%5D&cauthor=true&cauthor_uid=23984210). Coexisting Rathke cleft cyst and pituitary adenoma presenting with pituitary apoplexy: report of two cases. [*Skull Base Rep.*](https://www.ncbi.nlm.nih.gov/pubmed/23984210) 2011;**1**(2):99-104.
18. [Nishio S](https://www.ncbi.nlm.nih.gov/pubmed/?term=Nishio%20S%5BAuthor%5D&cauthor=true&cauthor_uid=3670583), [Mizuno J](https://www.ncbi.nlm.nih.gov/pubmed/?term=Mizuno%20J%5BAuthor%5D&cauthor=true&cauthor_uid=3670583), [Barrow DL](https://www.ncbi.nlm.nih.gov/pubmed/?term=Barrow%20DL%5BAuthor%5D&cauthor=true&cauthor_uid=3670583), [Takei Y](https://www.ncbi.nlm.nih.gov/pubmed/?term=Takei%20Y%5BAuthor%5D&cauthor=true&cauthor_uid=3670583), [Tindall GT](https://www.ncbi.nlm.nih.gov/pubmed/?term=Tindall%20GT%5BAuthor%5D&cauthor=true&cauthor_uid=3670583). Pituitary tumors composed of adenohypophysial adenoma and Rathke's cleft cyst elements: a clinicopathological study. [*Neurosurgery*.](https://www.ncbi.nlm.nih.gov/pubmed/3670583) 1987;**21**(3):371-7.
19. [Wang SS](https://www.ncbi.nlm.nih.gov/pubmed/?term=Wang%20SS%5BAuthor%5D&cauthor=true&cauthor_uid=23008711), [Xiao DY](https://www.ncbi.nlm.nih.gov/pubmed/?term=Xiao%20DY%5BAuthor%5D&cauthor=true&cauthor_uid=23008711), [Yu YH](https://www.ncbi.nlm.nih.gov/pubmed/?term=Yu%20YH%5BAuthor%5D&cauthor=true&cauthor_uid=23008711), [Jing JJ](https://www.ncbi.nlm.nih.gov/pubmed/?term=Jing%20JJ%5BAuthor%5D&cauthor=true&cauthor_uid=23008711), [Zhao L](https://www.ncbi.nlm.nih.gov/pubmed/?term=Zhao%20L%5BAuthor%5D&cauthor=true&cauthor_uid=23008711), [Wang RM](https://www.ncbi.nlm.nih.gov/pubmed/?term=Wang%20RM%5BAuthor%5D&cauthor=true&cauthor_uid=23008711). Diagnostic Significance of Intracystic Nodules on MRI in Rathke's Cleft Cyst. [*Int J Endocrinol*.](https://www.ncbi.nlm.nih.gov/pubmed/23008711) 2012;958732.
20. Francesco Belotti, Isabella Lupi, Mirco Cosottini, Claudia Ambrosi, Roberto Gasparotti, Fausto Bogazzi, Marco M. Fontanella, Francesco Doglietto. Persisting Embryonal Infundibular Recess (PEIR): Two Case Reports and Systematic Literature Review. J Clin Endocrinol Metab, 2018, 103(7):2424–2429
21. [Gao M](https://www.ncbi.nlm.nih.gov/pubmed/?term=Gao%20M%5BAuthor%5D&cauthor=true&cauthor_uid=26845092), [An Y](https://www.ncbi.nlm.nih.gov/pubmed/?term=An%20Y%5BAuthor%5D&cauthor=true&cauthor_uid=26845092), [Huang Z](https://www.ncbi.nlm.nih.gov/pubmed/?term=Huang%20Z%5BAuthor%5D&cauthor=true&cauthor_uid=26845092), [Niu J](https://www.ncbi.nlm.nih.gov/pubmed/?term=Niu%20J%5BAuthor%5D&cauthor=true&cauthor_uid=26845092), [Yuan X](https://www.ncbi.nlm.nih.gov/pubmed/?term=Yuan%20X%5BAuthor%5D&cauthor=true&cauthor_uid=26845092), [Bai Y](https://www.ncbi.nlm.nih.gov/pubmed/?term=Bai%20Y%5BAuthor%5D&cauthor=true&cauthor_uid=26845092), [Guo L](https://www.ncbi.nlm.nih.gov/pubmed/?term=Guo%20L%5BAuthor%5D&cauthor=true&cauthor_uid=26845092). The coexistence of Rathke cleft cyst and pituitary adenoma. J Craniofac Surg. 2016;27:e128-30.
22. Inder WJ, Macfarlane MR. Hyperprolactinaemia associated with a complex cystic pituitary mass: medical versus surgical therapy. Intern Med J. 2004; 34:573–576
23. J. J. Mukherjee, N. Islam, G. Kaltsas, D. G. Lowe, M. Charlesworth, F. Afashar, P. J. Trainer, J. P. Monson, G. M. Besser, A. B. Grossman. Clinical, Radiological and Pathological Features of Patients with Rathke’s Cleft Cysts: Tumors That May Recur. Journal of Clinical Endocrinology and Metabolism .1997; 82(7):2357-2362
