# Supplementary material for: A Potential Concomitant Sellar Embryonic Remnant-Associated Collision Tumor: Systematic Review
Source: Front Oncol. 2021 Apr 29;11:649958. doi: 10.3389/fonc.2021.649958 (PMC8117962; doi:10.3389/fonc.2021.649958)
Supplement: Supplementary file 4 [file DataSheet_4.docx]

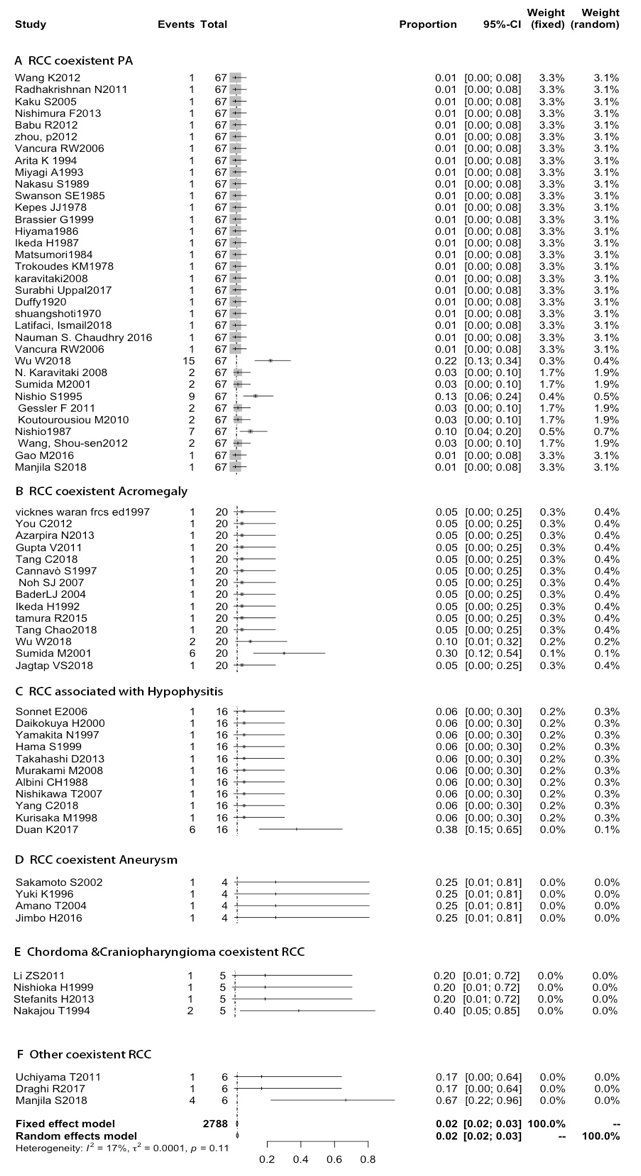


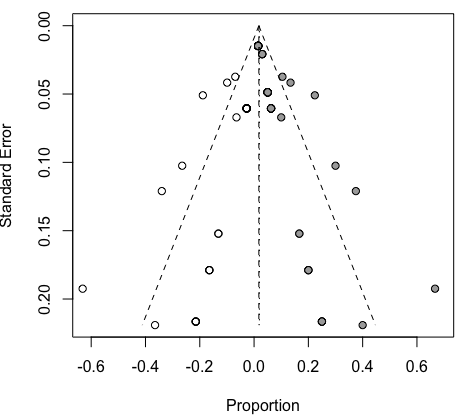


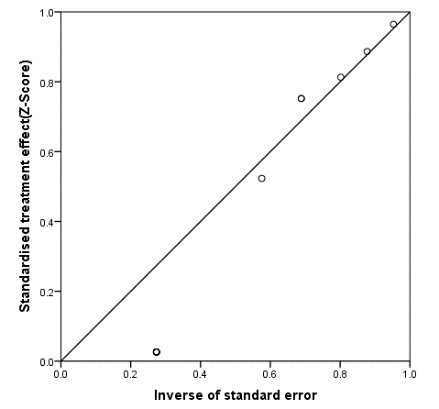


**Figure S1: Forest plots for the subgroup analysis of population–based RCC coexisting with various sellar lesion prevalence.**

(A): Forest plot of the subgroup analysis by RCC coexistent PA. (B) Forest plot of the subgroup analysis by RCC coexistent Somatotroph (Arcomegaly). (C) Forest plot of the subgroup analysis by RCC associated with Hypophysitis. (D) Forest plot of the subgroup analysis by RCC coexistent aneurysm. (E) Forest plot of the subgroup analysis by RCC coexistent Chordoma & Craniopharyngioma. (F) Forest plot of the subgroup analysis by RCC coexistent other (Epidermoid Cyst, Dermoid Cyst, pituitary granulomatosis, Salivary gland remnants. Epidermoid Cyst, Dermoid Cyst, pituitary granulomatosis, Salivary gland remnants)

Funnel plots, each point represents a separate study in the indicated association after 67 studies were filled by a nonparametric trim and fill method (the solid circle represent studies which were filled)

Linear regression test of funnel plot asymmetry (Kolmogorov-Smirnov Z test). The intercept indicating Normal distribution is 3.661. P-value = 0.000, indicating significant publication Normal distribution.
